# Part II: NiMoO_4_ Nanostructures Synthesized by the Solution Combustion Method: A Parametric Study on the Influence of Material Synthesis and Electrode-Fabrication Parameters on the Electrocatalytic Activity in the Hydrogen Evolution Reaction

**DOI:** 10.3390/molecules27041199

**Published:** 2022-02-10

**Authors:** Mahmoud Bassam Rammal, Vincent El-Ghoubaira, Sasha Omanovic

**Affiliations:** Department of Chemical Engineering, McGill University, 3610 University Street, Montreal, QC H3A 0C5, Canada; vincent.elghoubaira@mail.mcgill.ca (V.E.-G.); sasha.omanovic@mcgill.ca (S.O.)

**Keywords:** hydrogen evolution reaction, solution combustion synthesis, nickel molybdate, parametric study, water electrolysis, nanostructures

## Abstract

Earth-abundant NiMo-oxide nanostructures were investigated as efficient electrocatalytic materials for the hydrogen evolution reaction (HER) in acidic media. Synthesis and non-synthesis parameters were thoroughly studied. For the non-synthesis parameters, the variation in Nafion loading resulted in a volcano-like trend, while the change in the electrocatalyst loading showed that the marginal benefit of high loadings attenuates due to mass-transfer limitations. The addition of carbon black to the electrocatalyst layer improved the HER performance at low loadings. Different carbon black grades showed a varying influence on the HER performance. Regarding the synthesis parameters, a calcination temperature of 500 °C, a calcination time between 20 and 720 min, a stoichiometric composition (Ni/Mo = 1), an acidic precursor solution, and a fuel-lean system were conditions that yielded the highest HER activity. The in-house NiMoO_4_/CB/Nafion electrocatalyst layer was found to offer a better long-term performance than the commercial Pt/C.

## 1. Introduction

With an increasingly growing population and ever-lasting progress in technologies, the need for reliable and clean energy sources has never been of greater importance. Nevertheless, the dependence on fossil fuels to satisfy the mounting global energy demands has had deleterious effects on many aspects of life, including health complications, political conflicts, and oil price volatility, to name just a few. While eco-friendly alternatives like sun, wind, and hydro have been used for a relatively long time, their performance has been limited by the prevalent environmental conditions, a problem commonly referred to as intermittency [1]. Intermittent energy production is manifested by seasonal and daily fluctuations in the energy supply; this mismatch between supply and demand impedes the wide-scale commercialization of these sustainable systems, all but ensuring the continued predominance of fossil-fuel-based energy sources [2]. To address the intermittency problems associated with these renewable/sustainable energy systems, storing their excess energy in batteries and hydrogen gas (H_2_) is a viable option. While batteries are promising and have been gaining more attention with the explosive growth of companies that produce electric vehicles, they are expensive, voluminous, and are associated with toxic chemicals, low energy density, and longevity issues [3]. On the other hand, hydrogen gas (H_2_), which is made of the most abundant element in the universe and the lightest element in the periodic table (H), has been increasingly regarded as a promising energy vector in a future clean-energy landscape. Owing to its small molecular weight, H_2_ has the highest energy density per unit mass compared to any other available energy source/vector. It is an energy vector that can be used in a fuel-cell setup to produce electricity or can alternatively be utilized directly in an internal combustion setup with no undesirable emissions unlike carbon-based energy sources [4].

Currently, industries rely, in large part, on steam reforming to mass-produce H_2_ at an affordable price, whereby steam and hydrocarbons are reacted together at a high temperature to yield H_2_ alongside other harmful by-products (CO, CO_2_) [5]. In addition to this method’s high carbon footprint and non-sustainable nature, its efficiency is lackluster, and the produced H_2_ contains deleterious impurities, notably sulfur and carbon monoxide, which could deteriorate operations dependent on a high-purity H_2_ feedstock like fuel cells [6]. Among the presently available environmentally friendly H_2_ production alternatives (water electrolysis, biomass, photolytic splitting of water, and photobiological processes), water electrolysis is the most mature and viable option [7]. Water electrolysis, powered by renewable energies or surplus electricity in off-peak hours, is attracting a growing interest due to its potential of producing large amounts of sustainable H_2_ with a little environmental impact [8]. Despite the promising features of water electrolysis, its high cost remains a major challenge that must be addressed before a wide-scale commercialization strategy is adopted [9].

The high cost of water electrolysis is driven by many factors, including high energy consumption, performance degradation, use of expensive corrosion-resistant materials as catalytic materials, infrastructure limitations, and hydrogen storage difficulties [8]. An area where the cost can be reduced lies in the selection of the (electro)catalytic material [10]. Ni and Ni-based alloys are well-known catalytic materials used in the alkaline-based system (alkaline hydrogen electrolyzer); this system has long been utilized in the industry due to its established technology, reliability, good stability, and its reliance on inexpensive materials [11]. However, due to its restricted output (low current density), gas crossover, large footprint, and slow response time, another electrolyzer system has been developed to overcome these issues, leading to a revolutionary overhaul in the underlying technology [11]; that is the polymer electrolyte membrane (PEM) electrolyzer. Additionally, from a kinetic standpoint, the hydrogen evolution reaction (HER) activity of most electrocatalytic materials in the acidic environment, like that found in PEM systems, is about two to three orders of magnitude higher than that in the alkaline environment [12]. However, due to their harsh, corrosive environment, PEM electrolyzers rely on expensive and scarce platinum group materials (PGM) as electrocatalysts. In fact, platinum has been identified as the most active HER electrocatalyst [13]. Other relevant factors were also reported to be essential prerequisites for active HER materials, including but not limited to conductivity, crystallinity, surface area, and catalyst supports [14].

To bring PEM systems closer to commercialization, developing low-cost, earth-abundant electrocatalytic materials of high activity and stability (mechanical and electrochemical) across the whole pH scale is indispensable. Considering the volcano-plot model, the element that occupies the highest intrinsic activity after PGM is Ni. Nevertheless, Ni has a poor kinetic activity compared to PGM and is susceptible to corrosion in an acidic environment [15]. Alloying Ni with other metals of different electronic structures and hydrogen adsorption affinities can prompt a synergistic effect and bolster the electrode’s performance, despite lingering corrosion-related issues [16]. To circumvent the corrosion-related concerns plaguing Ni-based alloys, the use of transition-metal oxide materials (TMO) characterized with favorable chemical and mechanical stability has been proposed in water electrolysis [10]. TMOs are known to exhibit superior stability in harsh conditions, including in the acidic media—a feature that confers these materials with a favorable stability in PEM environments [17].

We have previously identified NiMoO_4_ as a promising HER electrocatalyst in the acidic medium [18] and reported on its synthesis as a nanostructured material [19]. In Part I of the current manuscript [20], we performed a detailed study on the production, optimization, and characterization of NiMoO_4_ nanostructures using the solution combustion synthesis (SCS) method, with a comprehensive assessment of several synthesis-related parameters (the effects of calcination temperature, calcination time, Ni/Mo ratio, pH of the precursor solution, and fuel-to-oxidant ratio (φ)) that could influence the microstructure and physicochemical properties of the material. In the current (Part II) study, we evaluate the electrocatalytic activity of the nanostructured NiMoO_4_ samples (synthesized and characterized in Part I [20]) toward HER in an acidic environment (resembling the one in a PEM electrolyzer) to deduce the influence of material production parameters on the HER activity. Since HER cathodes in PEM electrolyzers are typically comprised of nanostructured metal electrocatalyst materials usually anchored on carbon black (CB) particles, and since the ionomer (Nafion) is a key part of the PEM configuration, we also elucidate here the effect of several other non-synthesis influential parameters related to the makeup of the electrocatalyst layer (Nafion loading, electrocatalyst loading, CB loading, and CB type) aiming to pinpoint the set of conditions that yield the best HER performance. This comprehensive study also compares the HER performance of our optimized (best-performing) NiMoO_4_/Nafion/CB layer to the current state-of-the-art commercial HER electrocatalyst (Pt/C) commonly used in PEM electrolyzers. Finally, we discuss the in-house electrocatalyst’s mechanical and electrochemical stability and assess the long-term HER performance of NiMoO_4_ relative to the commercial Pt/C and other benchmark materials.

## 2. Results and Discussion

The following sections will discuss the influence of various parameters (non-synthesis and synthesis) on the HER performance of an electrocatalyst layer consisting of NiMo-oxide powder, CB, and Nafion, with the aim of understanding the role of these experimental parameters on the HER electrocatalytic activity.

### 2.1. Non-Synthesis Parameters

#### 2.1.1. Effect of Nafion Loading

In PEM water electrolyzers, the anode and cathode electrocatalysts are pressed against a proton-exchange membrane (PEM), typically a commercial Nafion membrane, to form a membrane-electrode-assembly (MEA). Nafion in PEM electrolyzers offers excellent H^+^ ionic conductivity, low gas crossover, compact density, and good electrical insulation, which allows for higher current and voltage operations [6]. In addition to providing proton conductivity, Nafion incorporated into the electrocatalyst layer also serves as a binder, imparting the electrocatalyst layer with mechanical stability and better durability. Therefore, it is important to optimize the amount of Nafion in the electrocatalyst layer to achieve the right balance between proton and electron conductivity while ensuring the structural integrity of the catalyst. However, research on optimizing the amount of Nafion incorporated into the electrocatalyst layer of PEM water electrolyzer cathodes has been scarce. To date, only a very few studies, most of which focus on fuel-cell systems, have examined this topic. Therefore, more research is warranted to better explain the effect of Nafion loading on the HER performance, especially in the context of the current research that is based on the use of metal-oxide-based cathodes, rather than platinum-based cathodes [21].

**Figure 1:**
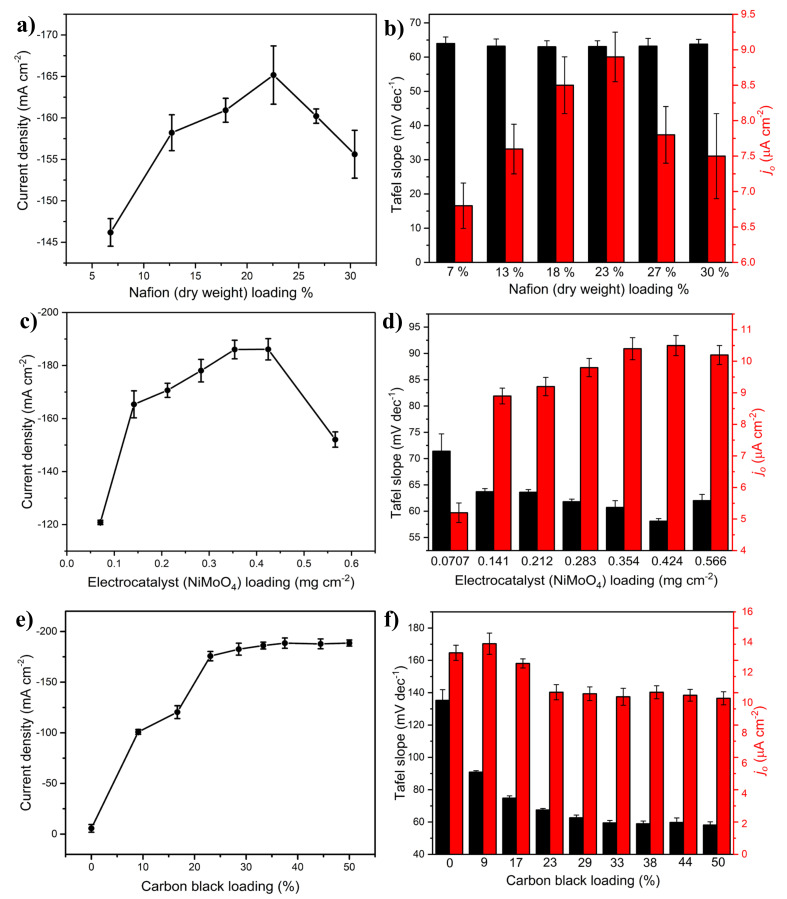
Dependence of the HER current density on the (**a**) Nafion loading, (**c**) electrocatalyst (NiMoO_4_) loading, and (**e**) carbon black loading in the electrocatalyst layer consisting of NiMoO_4_, carbon black, and Nafion. The current density values were recorded under potentiostatic conditions at an overpotential of −0.58 V after reaching a quasi-steady-state HER rate (1000 s). This was followed by LSV curves at a scan rate of 1 mV s^−1^ and corrected for ohmic (*iR*) drop, shown in Figure S1a,c,e. The variation in Tafel slopes and exchange current density for the (**b**) effect of Nafion loading, (**d**) electrocatalyst (NiMoO_4_) loading, and (**f**) carbon black loading. Those parameters were obtained from the Tafel plots shown in Figure S1b,d,f. In Nafion testing: the loading of NiMO_4_ was 0.141 mg cm^−2^, while the amount of CB was 33 wt.% relative to the total weight of the NiMoO_4_/CB mixture. In the electrocatalyst loading tests, the Nafion amount was 23 wt.%, and the amount of CB was 33 wt.%. In the CB loading test, the Nafion amount was 23 wt.%, while the catalyst loading was 0.283 mg cm^−2^.

Figure 1a illustrates the effect of Nafion loading on the HER performance of the electrocatalyst layer. A volcano-like trend is evident, in which the best HER performance occurred at ca. 23% Nafion (dry weight basis). On the left side of the summit, the performance was enhanced with the incremental increase in the amount of Nafion. The poor HER performance observed at low Nafion loadings can be attributed to the slight delamination of the catalytic layer, as manifested by the presence of a considerable number of catalyst-layer particles in the electrolyte after electrolysis due to the compromised mechanical stability of the electrocatalyst layer. Additionally, at low Nafion loadings, the overall low ionic (proton) conductivity of the electrocatalyst layer presents another origin of the poor HER electrocatalytic activity of the layer. As the amount of Nafion in the electrocatalyst layer increased, the mechanical integrity of the electrocatalyst layer improved, and so did its proton conductivity, yielding the best HER performance at ca. 23 wt.%. Beyond this, to the right of the summit, the performance decreased as the electrical conductivity of the electrocatalyst layer became the limiting factor due to the electrically insulating nature of Nafion, limiting the electrical conductivity of the nanostructured NiMoO_4_ active species. The decline in performance at high Nafion loadings can be attributed to mass-transfer limitations associated with the decreased porosity of the layer, causing a slower outbound flux of the formed hydrogen bubbles. The optimized Nafion loading (23 wt.%) obtained in this study is slightly lower than what has been reported as an optimal Nafion loading in fuel-cell applications [21] but within the range that has been found for commercial PEM electrolyzers based on Pt cathodes (20 to 30 wt.% of the dry electrocatalyst weight) [6].

The electrochemical production of H_2_ proceeds through a multi-step pathway on the surface of the electrocatalyst. The most widely accepted mechanism for HER in the acidic medium constitutes a two-stepped route. In Equation (1) below, known as the discharge reaction (Volmer step), an interaction occurs on an active site of the electrocatalyst surface (M) between a proton from the electrolyte and an electron from the external circuit, resulting in an adsorbed hydrogen atom (H_ads_).

Step I: H^+^_(aq)_ + M + e^−^ → M-H_ads_ (1)

Subsequently, hydrogen desorption may proceed to form molecular H_2_ through one of two subsequent steps. In one pathway (Equation (2)), commonly known as the electrochemical desorption step (Heyrovsky reaction), H_ads_ adsorbed on the electrocatalyst surface (Equation (1)) reacts with another proton from the solution, concomitant with a second electron transfer to form molecular hydrogen.

Step II (a): H^+^_(aq)_ + M-H_ads_ + e^−^ → H_2__(g)_ + M (2)

Alternatively, in the other possible pathway (Equation (3)), a recombination step (Tafel reaction) could take place in which two H_ads_ adsorbed on two adjacent electrocatalyst sites combine to generate molecular hydrogen.

Step II (b): M-H_ads_ + M-H_ads_ → H_2__(g)_ + 2M (3)

Tafel plots (Figure S1) can be used to determine which of the above three steps is rate-determining in the HER, in addition to enabling the determination of the exchange current density. Thus, the slope of the Tafel curves in the linear region in Figure S1 gives insight into the HER mechanism, while the value of the current at zero overpotential, obtained by extrapolating the Tafel line, yields the exchange current density (*j_o_*). *j_o_* describes the reaction rate at equilibrium conditions and is used widely in the literature to assess the “basic/initial” electrocatalytic activity of HER electrocatalysts. With regards to the above-outlined HER steps (Equations (1)–(3)), the corresponding theoretical Tafel slopes at standard conditions were 120, 40, and 30 mV dec^−1^, respectively.

Figure S1b shows the Tafel plot pertinent to the effect of Nafion loading. By polarizing the electrocatalysts to more negative HER overpotentials, the Tafel curve first displays a linear region, thus evidencing that the HER within this potential region is electron-transfer controlled. However, at overpotentials larger than ca. −0.2 V, the curve deviates from linearity, which indicates that other processes influence the HER kinetics. In this case, it is most likely that the deviation from linearity was due to the blockage of the NiMoO_4_ active sites by H_2_ bubbles, the degree of which increased with overpotential.

Figure 1b shows that the Tafel slope values (black bars) for all the studied samples exhibited almost the same value (~65 mV dec^−1^), indicating that the HER mechanism associated with the samples of varying Nafion loadings remained the same: the RDS in the HER associated with these samples was likely the Heyrovsky step, even though the experimental values are higher than the corresponding theoretical value (40 mV dec^−1^). This is reasonable for metal oxides and electrocatalyst layers like those synthesized in this study, consisting of multiple components (NiMoO_4_/CB/Nafion). In addition, mass-transport limitations in these structures can influence the Tafel slope both in terms of proton access to the electroactive sites and the local pH variations. Moreover, the presence of CB might play a role in the HER mechanism (discussed in detail in the following section).

On the other hand, the Nafion loading influences the exchange current density (red bars), which follows the same trend as that presented in Figure 1a; the highest exchange current density value was obtained at a Nafion loading of 23 wt.%.

#### 2.1.2. Effect of Catalyst (NiMoO_4_) and Carbon Black Loading

The cathode material represents a considerable portion of the total PEM electrolyzer cost as degradation/corrosion plagues almost any non-noble material in the harsh, acidic environment like that present in PEM systems, even when the electrolyzer is in an open-circuit mode. Today, loadings for the cathode side range between 0.5 and 1 mg cm^−2^ for commonly used electrocatalysts (usually Pt), and further reductions will always be demanded in a broad cost-reduction strategy [11].

Although the NiMoO_4_ electrocatalyst used in this work is considerably cheaper than the Pt-based electrocatalysts commonly used in commercial PEM electrolyzers, it was of interest to optimize its loading in the electrocatalyst layer, as shown in Figure 1c. As the electrocatalyst loading increased, the HER performance of the cathode improved, reaching a maximum at a loading of ca. 0.4 mg cm^−2^. For illustration purposes, a side-view representative image portraying the electrocatalyst (loading: 0.343 mg cm^−2^) is shown in Figure S2a, while a profilometer measurement of the catalyst thickness is portrayed in Figure S2b. With a further increase in the electrocatalyst loading to 0.566 mg cm^−2^, its HER electrocatalytic activity degraded due to the excessive thickness of the electrocatalyst layer and the resulting mass-transfer barriers [22]. Interestingly, it was notable that at this high loading (0.566 mg cm^−2^), the electrocatalyst layer delaminated shortly after electrolysis, lowering the total loading of the electrocatalyst. Additionally, with the increase in the electrocatalyst/CB ratio at higher NiMoO_4_ loadings, agglomeration of the electrocatalyst can become pronounced, thereby lowering the total active surface area available for the HER (the effect of CB on the electrocatalyst agglomeration is discussed further in Section 2.1.3). 

Gray et al. [16] noticed a similar loading-dependent behavior in the HER using a NiMo-powdered electrocatalyst, establishing a power-law relationship between the HER current density and the mass loading of the electrocatalyst. The authors reported a progressive decline in the activity at increasing film thickness (higher loadings) due to mass-transfer limitations. Figure 1c also shows a larger relative increase in the HER current going from the lowest to the second-lowest loading of the electrocatalyst (0.0707 to 0.141 mg cm^−2^), followed by a slower, gradual increase. In theory, doubling the mass loading of NiMoO_4_ should have resulted in doubling the electrocatalyst’s surface area and thus the HER current; this is not reflected in Figure 1c. However, one should bear in mind that increasing the electrocatalyst loading can also increase the electrocatalyst layer’s thickness, restricting the access of H^+^ to NiMoO_4_ active sites and bringing about mass-transport limitations. These negative effects seem less pronounced at the first two NiMoO_4_ loadings (0.0707 and 0.141 mg cm^−2^) due to the smaller electrocatalyst layer thickness, thus explaining the larger relative increase in HER current than what was recorded at higher electrocatalyst loadings.

Figure 1d shows the dependence of Tafel slopes on the electrocatalyst loading. The trend follows that shown in Figure 1c, where the largest drop in the Tafel slope occurred when going from the lowest to the second-lowest electrocatalyst loading, proceeding by a gradual decrease until reaching a minimum at a loading of 0.242 mg cm^−2^, followed by a slight increase at higher loadings. Although the obtained Tafel slopes are in a range that indicates the Volmer–Heyrovsky mechanism, the observed trend implies that the HER mechanism varies slightly with the electrocatalyst loading. The exchange current density (Figure 1d) follows the same trend as that seen for the HER performance measured at a constant overpotential, Figure 1c. Regarding the exchange current density, a drastic increase (Figure 1d) transpired upon increasing the electrocatalyst loading from 0.0707 to 0.141 mg cm^−2^ after which the rate of change of the exchange current density appeared to level off with higher electrocatalyst loadings until 0.566 mg cm^−2^ where it declined.

NiMoO_4_ is a semiconductor, thus offering lower electrical conductivity than Pt and other noble-metal cathode electrocatalysts used in the PEM water electrolyzers. To circumvent this issue, the NiMoO_4_-based electrocatalyst layer should be supplemented with a conductive additive. CB is an inexpensive, readily available, and effective conductive material that has been used in PEM electrolyzers, fuel cells, Li-ion batteries, and supercapacitors, to mention just a few [23,24]. Vulcan XC-72R is the most popular CB type utilized in electrochemical applications because of its good balance between electrical conductivity and surface area [25]. The necessary amount of CB (loading) that must be added into a catalyst/electrode layer to achieve the optimal performance depends on the desired application and the nature of the electrocatalyst. Therefore, the effect of the CB loading (Vulcan XC-72R) on the HER performance of the NiMoO_4_ electrocatalyst layer was investigated, and the results are discussed as follows.

As shown in Figure 1e, in the absence of CB, the HER performance of NiMoO_4_ was negligible, due to the relatively poor electrical conductivity of the catalyst. As a small amount of CB was added (ca. 9 wt.%), a significant improvement in the HER (a 20-fold increase in the current density) was observed. With higher CB loadings, additional enhancement in the HER performance occurred, until leveling off at a CB loading of ca. 30 wt.%. It is to be noted that pristine Vulcan XC-72R is not an active material for HER, and its function is limited to enhancing the electrical conductivity of the catalytic material (NiMoO_4_). Hence, the increase in the HER kinetics at high CB loadings, observed in Figure 1e, can be ascribed to several factors.

Firstly, NiMoO_4_, in both its common phases (alpha and beta), is a semiconductor with a lower electrical conductivity compared to metal conductors [26]. Since HER is an electrochemical process involving electron transfer, the lower conductivity of the NiMoO_4_ electrocatalyst presents a limiting factor, resulting in a potential drop across the NiMoO_4_ electrocatalyst layer and thus in an increased HER overpotential. Due to its good electrical conductivity, adding CB into the NiMoO_4_ electrocatalyst layer resulted in an enhancement in the overall conductivity of the electrocatalyst layer, ultimately decreasing the HER overpotential and improving its kinetics, as demonstrated in Figure 1e. Initially, a sharp increase (ca. two orders of magnitude) in HER activity occurred upon adding ca. 9 wt.% of CB to NiMoO_4_. This large relative increase can be attributed to the formation of an electronically conducting network of CB particles. A further increase in CB loading yielded a smaller relative increase in HER activity, eventually leveling off at ca. 30 wt.%. Indeed, the results here combined with other observations in the literature point to the existence of a percolation threshold for enhancing the electrocatalytic performance, beyond which further additions are not beneficial to the HER activity [25,27].

Although the mechanism by which CB influences the conductivity of the electrocatalyst has been rarely studied in HER-related works focused on metal-oxide-based electrocatalysts, the classical percolation theory can be used to describe the trend in Figure 1e. NiMoO_4_ is a semiconductor, and as such, the principle of the percolation method, in our opinion, could be applied here to understand the progression of the electrical conductivity in the electrocatalyst layer upon adding CB to it. In this theory, the gradual addition of conductive carbon particles into an insulating matrix leads to what is known as the “critical concentration” at which the percolation occurs [27]. Percolation represents the transition between the insulating and the conductive zones, beyond which further CB addition does not impact the conductivity (conduction zone). The pronounced increase in the HER performance (Figure 1e), observed upon utilizing a low loading of CB, points to the existence of a percolation threshold between 0–10 CB wt.%. This agrees with the findings of Dominko et al. [28], who noted that 3 wt.% CB was sufficient to significantly improve the electrical conductivity of LiFeO_4_ composite cathodes.

Another important factor related to the use of CB concerns the change in the surface area of the catalyst (NiMoO_4_) with different CB loadings. The addition of CB can reduce the aggregation of nanostructured catalysts (that are generally prone to aggregation due to their high surface energy) and enhance the use of the active sites, allowing a rapid charge transfer [23,29]. The BET surface area of NiMoO_4_ is ca. 26 m^2^/g [19], while that of pristine Vulcan XC-72R is ca. 238 m^2^/g [24]. A notable increase in specific surface area and pore volume was observed upon a small addition of CB (10 wt.%) to NiMoO_4_, as shown in Table 1. With the CB addition, the total surface area of the NiMoO_4_/CB mixture was higher than the sum of the constituents, a clear indication that agglomeration of NiMoO_4_ was alleviated with the use of CB [30].

In addition, the presence of CB in the electrocatalyst layer could play a role in the HER mechanism by providing favorable adsorption sites and serving as an electron sink. This has been corroborated by Joshi et al. [31], who postulated a two-site model for the synergistic effect between the electrochemically active material and CB. The mechanism is based on the well-known spill-over theory involving a nonelectrochemical step in which CB serves as an electron sink mediating the diffusion of the adsorbed hydrogen (H_ads_) between the electroactive species. Therefore, for the in-house NiMoO_4_-based electrocatalyst, it can be envisaged that the HER occurs as follows: In the first step (Volmer), H^+^ gets adsorbed on Ni that has a good adsorption affinity and thus acts as a source of H. H_ads_ then diffuses onto the carbon particles (nonelectrochemical step) and eventually onto the neighboring Mo sites where the combination of H_ads_ with a proton from the electrolyte takes place leading to H_2_ production (see Equations (1) and (2)) [15]. In fact, previous reports also indicated that delocalized π-electrons of graphitic layers present in CB could act as Lewis-base elements promoting the adsorption of protons from the solution and resulting in an improved catalytic performance [32]. The premise that CB participates in the HER mechanism could be supported by the change in Tafel slopes as a function of CB loading, as seen in Figure 1f. Without CB in the electrocatalyst layer, the Tafel slope indicates that the Volmer step was the RDS. However, as the amount of CB increased, the Tafel slope experienced a marked drop, indicating a shift in the RDS to the Heyrovsky step and therefore the prevalence of the Volmer–Heyrovsky mechanism. The value of the Tafel slope when CB was used with NiMoO_4_ is indeed similar to that reported in previous reports [22,31], where CB was either used as a support or a conductive element. However, despite its contribution to the mechanism change, it should be noted that the role of CB was limited to increasing the electrical conductivity and enhancing the kinetics of the reaction but was not kinetically active on its own toward the HER, as it will be seen in Section 2.3 later in the text.

#### 2.1.3. Effect of Carbon Black Type

**Figure 2:**
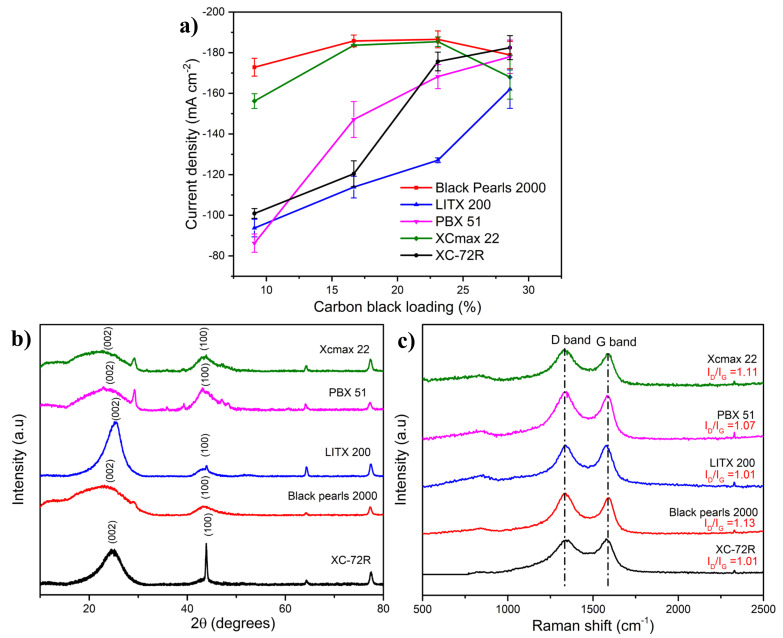
(**a**) Dependence of the HER current density on the carbon black type used in the electrocatalyst layer consisting of NiMoO_4_, carbon black, and Nafion. The corresponding NiMoO_4_ and Nafion loadings were 0.283 mg cm^−2^ and 23 wt.% (dry weight), respectively. The current density values were obtained from a potentiostatic test conducted at −0.58 V in 0.5 M H_2_SO_4,_ after reaching a quasi-steady state HER rate (1000 s). (**b**) XRD. (**c**) Raman spectra of the pristine CB types.

While CB loading is an essential parameter in influencing the electrical conductivity, and in turn, the electrocatalytic performance toward the HER, the type of CB should also be considered in this framework. CB exists in various commercial types and is manufactured by different processes and feedstocks, thus resulting in a wide range of physicochemical properties [33]. The physical and chemical properties of CB could greatly influence its conductivity and other physicochemical properties (e.g., specific surface area and pore volume) and ultimately the HER performance of the catalyst to which it is added. Therefore, the proper selection of a suitable CB is critical for a successful end-use performance. In this section, we discuss the effect of CB type on the HER performance, aiming to elucidate the effect of the CB properties on its function as a conductive additive to the NiMoO_4_ electrocatalyst layer—an aspect that has rarely been addressed previously. Different types of CB were tested (Figure 2) including: (1) Vulcan XC-72R, utilized frequently as a catalyst support in the proton-exchange membrane fuel cells (PEMFC); (2) Vulcan XCmax 22, which has been recently commercialized as a superconductive CB additive, in addition to CBs used in other applications such as; (3) Black Pearls (BP) 2000, which is a high-surface-area CB commonly utilized in rubber-based devices for static dissipation; (4) LITX 2000, which is used as a specialty carbon in Li-ion batteries to provide high power and energy density; and (5) PBX 51, which is often utilized in lead-acid batteries for a superior dynamic-charge acceptance.

Figure 2a shows the effect of CB type on the HER performance of the NiMoO_4_-based electrocatalyst layer. At a low CB loading (9 wt.%), the best HER performance was achieved with BP 2000, followed closely by XCmax 22. The remaining CB grades (XC-72R, LITX 200, and PBX 51) exhibited a relatively poor HER activity at the same loading. At higher CB loadings, all the studied CBs achieved a marked increase in performance, although BP 2000 and XCmax 22 showed a relatively lower rate of increase. It is worth noting that as the CB loading approached 29 wt.%, all the investigated CBs attained the same limiting level of current density, except LITX 200, which remained slightly lower than the rest of the CBs. It is also notable that XCmax 22 and BP 2000 presented a slight decline in performance at the highest studied loading (29 wt.%) for reasons that will be discussed later in the text.

Different CB grades can impart different levels of conductivity, at a given loading, due to the various physical properties CBs can possess. Previous studies [34] have indicated that different CB grades present varying percolation thresholds, and therefore the electrical-conductivity–loading relationship is intimately linked to the physicochemical properties of the employed CB type. Additionally, apart from the electrical conductivity, some other characteristic features of CB could prove useful in enhancing the HER performance of the electrocatalytic material: structure, particle size, and surface chemistry stand out as the most essential ones.

The structure is one of the most important attributes of CBs and designates the number of particles per aggregate and the size and shape of those aggregates [35]. In fact, the structure is a measure of the CB aggregate complexity and describes the way constituent primary particles are connected and their void volume. Highly structured CBs have many primary particles and considerable branching and chaining, providing more potential paths for electron transfer. To quantitively evaluate the structure of CB, the oil absorption number (OAN) can be used, which refers to a universal standard test method (ASTM D2414) that describes the ability of a CB to absorb liquid (dibutyl phthalate or paraffin oil) [35]. CB grades with an OAN higher than 170 are classified as conductive CB, indicating a generally more complex structure. Relating the OAN of the studied CBs (Table 2) to their HER activity (Figure 2a) shows that the CB grades of higher OAN exhibit the best HER performance. The observations obtained here establish that for metal oxides of a semiconductor nature, like NiMoO_4_, the selection of an appropriate CB should strongly consider the OAN to achieve the best HER performance at the lowest CB loading.

The specific surface area/particle size is another important factor in assessing the effectiveness of conductive CB grades. CBs with smaller particle sizes result in more aggregates per unit weight. This introduces smaller distances between aggregates and promotes electron transfer from one aggregate to another. The specific surface area and pore volume of the pristine CBs assessed in this work are found in Table 2 [23,36]. The CB with the highest specific surface area was BP 2000, followed by PBX 51 and XCmax 22, and far behind existed the low-surface-area types (XC-72R and LITX 200). To better understand the interaction between CB and the NiMoO_4_ in the electrocatalyst layer, the surface area and pore volume of the NiMoO_4_/CB mixture (9 wt.% CB loading) were measured, and the values are presented in Table 2. Upon mixing NiMoO_4_ with a small amount of CB, the surface area of the mixture declined compared to that of pristine CB, which is due to the significant difference between the specific surface area of CB and that of NiMoO_4_ (Table 1). High-surface-area CB can play a role in the electrochemical performance by lowering the agglomeration/aggregation of the NiMoO_4_ electrocatalytic nanoparticles, thus enabling more accessible active electrocatalyst sites for hydrogen production [37]. However, when comparing the data in Table 2 and the HER results in Figure 2a for the same CB loading, it can be observed that neither the specific surface area of pristine CB nor that of the NiMoO_4_/CB mixture seem to be the overarching factor, since PBX 51 exhibited the poorest HER performance, yet its surface area was higher than that of XCmax 22, XC-72R, and LITX 200. On the other hand, the HER performance appeared to be more influenced by the porosity of the NiMoO_4_/CB mixture (Table 2), which much closely correlates with the HER performance trend observed in Figure 2a. Since the porosity is closely associated with the structure of CB, the results indicate that the CB structure is more impactful on the conductivity and HER activity of the electrocatalyst than the CB’s specific surface area/particle size.

Surface chemistry is another key property to consider in electrically conductive CBs [38] and is indicative of defects or CB surface functional groups (e.g., carboxyl, carboxylate, ether, quinine, and phenol). It has been suggested that the presence of oxygen-based functional groups could play a role in forming an insulating barrier and thereby reducing the conductivity of CB [39]. To characterize the surface chemistry of CBs used here, XPS analysis was conducted, and the resulting spectra are shown in Figure S3. The corresponding amounts of C, O, and S present in the CBs are listed in Table 3. Comparing the two best-performing CBs, XCmax 22 and BP 2000, it is notable that their XPS spectra are highly similar with practically identical amounts of C, O, and S. XC-72R, on the other hand, contained the lowest comparative amount of oxygen, while PBX 51 had a low oxygen content and a minor sulfur amount. LITX 200 had the highest amount of oxygen-rich functional groups. Since the amount of non-carbon elements in CB does not seem to correlate with the HER performance (Figure 2a), it is reasonable that the presence of oxygen and sulfur functional groups on the CB surface does not have a clear impact on their electrical conductivity and the HER performance, in agreement with previous findings [40]. However, the presence of surface functional groups can play a positive role in enhancing the wettability and dispersion of the CB particles, thus improving the HER kinetics [24].

**Table 2:** Specific surface area, pore volume, oil absorption number (OAN), and conductivity index of the studied pristine CB types. BET surface area and pore volume of a NiMoO_4_/CB mixture with different CB types and a CB loading of 9 wt.%.

Material	BET * Surface Area (m^2^/g)	BET Surface Area of NiMoO_4_/CB Mixture with 9 wt.% CB (m^2^/g)	Pore Volume NiMoO_4_/CB Mixture with 9 wt.% CB (cm^3^/g)	OAN **	Conductivity Index
XC-72R	234	61.6	0.182	175	120
Black Pearls 2000	1580	332.3	0.682	330	146
LITX 200	159	42.9	0.154	162	21
PBX 51	1420	237.2	0.275	170	119
XCmax 22	1360	303.7	0.525	320	142

* The BET surface area of Black Pearls 2000, LITX 2000, PBX 51, and XC-72R were obtained from [41] and that of XCmax 22 from [42]. ** The OAN numbers (mL (100 g)^−1^) of LITX 200, PBX 51, and XCmax 22 were obtained from the supplier’s (Cabot) data brochures, while those of Black Pearls 2000 and Vulcan XC-72R were based on [35].

A simple correlation that encompasses the effect of the previously mentioned factors (structure, particle size, and surface chemistry) on the electrical conductivity of CB is the conductivity index, shown in Equation (4) [43]:

(4) $$\mathrm{Conductivity}\;\mathrm{index}=\;\frac{{\left(\mathrm{BET}\;\mathrm{surface}\;\mathrm{area}\;\times \;\mathrm{OAN}\right)}^{1/2}}{\left(1\;+\;\mathrm{volatile}\;\mathrm{content}\right)}$$

where the “volatile content” represents the amount of surface oxygen groups and can be approximated from the XPS analysis (Table 3, Figure S3). Thus, a lower surface area (BET) and a lower structure (OAN) would result in a lower conductivity index and subsequently a higher electrical resistance, whereas a lower volatile content would entail a higher conductivity index. Black Pearls 2000 and XCmax 22 exhibited the highest conductivity indexes owing to their high surface area and OAN (Table 2) while exhibiting low volatile content. The conductivity indices were calculated for all the CB types utilized in this work (Table 2), where the results demonstrate that the HER activity in Figure 2a strongly correlates with the electrical conductivity of CB [43].

The level of the graphitic component in CB has also been reported to be an important characteristic feature that is a function of the CB type [44]. To investigate this aspect, the graphitic nature of the utilized CBs was probed using XRD and Raman spectroscopy. The XRD spectra in Figure 2b illustrate that the studied CBs exhibited different levels of crystallinity, where XC-72R and LITX 200 showed the highest crystallinity with similar profiles, compared to the high-surface-area CBs (XCmax 22, BP 2000, and PBX 51), which displayed broader peaks. Diffraction peaks centered at ~25° and 43.5° correspond to (002) and (100) crystallographic planes in carbon [41]. The relative intensity of (002) is reflective of the CB type graphitization degree and increased in the following order in Figure 2b: LITX 200 > XC-72R > PBX 51 > BP 2000 ≅ XCmax 22, but this does not coincide with the HER performance trend seen in Figure 2a.

Raman spectroscopy is another useful method to evaluate the graphitization of CB by assessing the G band at ~1335 cm^−1^ (graphite) and D band at ~1585 (sp^2^ carbon-induced spectra) of carbon-based materials. The relative intensity of these two bands (I_D_/I_G_) yields information about the graphitic-layer structure and the edge plane or boundaries of the graphitic crystal faces found in CB. Higher I_D_/I_G_ indicates a defect structure degree, while a lower ratio signifies a higher degree of graphitization [45]. Figure 2c shows that the degree of graphitization increased (I_D_/I_G_ decreased) in the order of LITX 200 ≅ XC-72R > PBX 51 > BP 2000 ≅ XCmax 22, which is a similar trend to that obtained with the XRD spectra (Figure 2b). While previous works presented contradictory findings regarding the role of I_D_/I_G,_ with some studies claiming that lower I_D_/I_G_ leads to better conductivity and electrochemical performance [40], we did not observe such an occurrence in our work. 

In conclusion, this section demonstrated that the proper selection of the CB type cannot be assessed by looking at one factor, but several physicochemical properties must be considered; however, the conductivity index, which encompasses the influences of several physicochemical properties (Table 2), strongly correlates with the HER performance observed in this study. 

### 2.2. Synthesis Parameters

After investigating the effect of non-synthesis parameters on the HER performance of the electrocatalyst layer, the effect of synthesis parameters used in the production of NiMoO_4_ (effects of the calcination temperature, calcination time, Ni/Mo atomic ratio, pH of the precursor solution, and fuel-to-oxidant ratio (φ)) on the HER performance are discussed below. Those non-synthesis parameters were studied extensively in Part I of this article [20]. Unless being the parameter studied, the calcination temperature and time were 500 °C and 6 h, respectively, the Ni/Mo atomic ratio was 1, the pH of the precursor solution was 4.57, and the fuel-to-oxidant ratio (φ) was unity. The non-synthesis parameters utilized to produce the electrocatalyst layer were: 23 wt.% (dry weight) of Nafion, a NiMoO_4_ electrocatalyst loading of 0.283 mg cm^−2^, a CB type and loading of XC-72R and 38 wt.%, respectively.

#### 2.2.1. Effect of Calcination Temperature and Time

**Figure 3:**
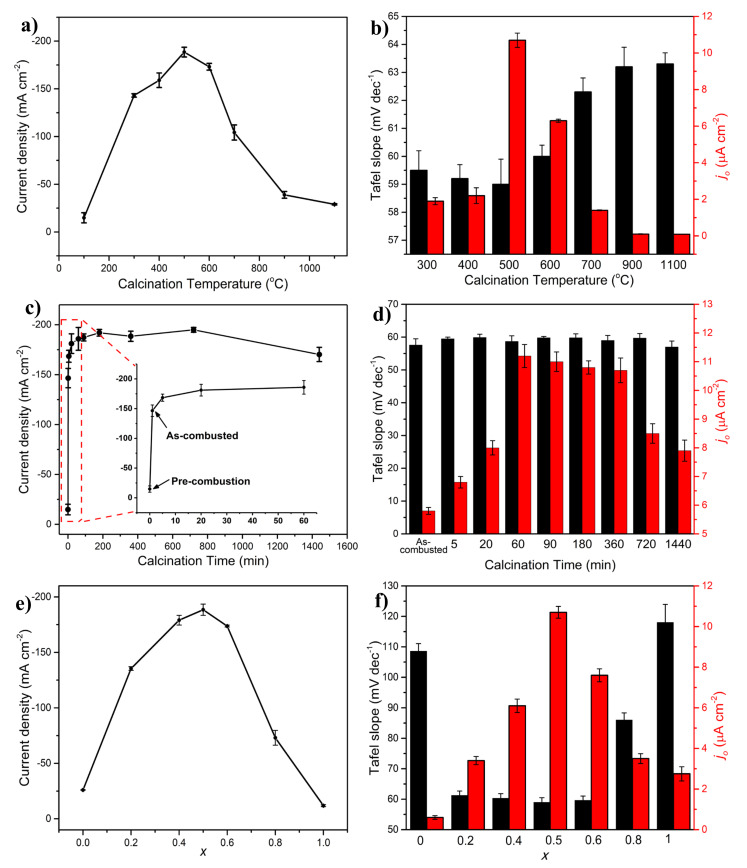
Dependence of the HER current density on the (**a**) calcination temperature, (**c**) calcination duration, and (**e**) Ni/Mo atomic ratio in Ni*_x_*Mo_1−*x*_-oxide (0 ≤ *x* ≤ 1). For (**a**,**c**), the electrocatalyst layer consisted of NiMoO_4_, carbon black, and Nafion while for (**e**) the electrocatalyst layer consisted of Ni*_x_*Mo_1−*x*_-oxide (0 ≤ *x* ≤ 1), carbon black, and Nafion. The current density values were recorded under potentiostatic conditions at an overpotential of −0.58 V after reaching a quasi-steady-state HER rate (1000 s). This was followed by LSV curves at a scan rate of 1 mV s^−1^ and corrected for ohmic (*iR*) drop, shown in Figure S1g,i,k. The variation in the Tafel slope and the exchange current density for the (**b**) effect of calcination temperature, (**d**) calcination time, and (**f**) Ni/Mo atomic ratio in Ni*_x_*Mo_1−*x*_-oxide; these kinetic parameters were obtained from the Tafel plots presented in Figure S1h,j,l, respectively.

The calcination temperature carries significant implications on the physicochemical features of the electrocatalyst; this parameter can greatly impact the electrocatalytic performance in applications such as the HER [46]. The HER performance of the NiMoO_4_-based electrocatalyst layer, prepared at different calcination temperatures, is shown in Figure 3a. The HER trend exhibits a volcano-like behavior, where the summit occurs at 500 °C, and the trend can be described as follows.

In our previous publication, we asserted that in the synthesis process of NiMoO_4_, a combustion reaction transpires at an ignition temperature of ~147 °C, sweeping through the precursor material and producing a fluffy, foamy product in a few seconds [19]. Below this temperature, the material (mostly made of precursor material at this stage) was amorphous, thus yielding a negligible activity toward the HER at 100 °C, as seen in Figure 3a. At 300 °C, the electrocatalyst’s performance in the HER saw a significant increase due to the crystallization and formation of NiMoO_4_ as a result of the combustion reaction. As it was shown in Part I of this article [20], XRD and FTIR analyses of the material produced at 300 °C indicated the presence of a wide range of phases, including MoO_2_, MoO_3_, β-NiMoO_4_, α-NiMoO_4_, and NiO. Additionally, the BET (specific) surface area was not maximized at this temperature, and the band gap, which can be correlated with the electrical conductivity, was relatively small (2.88 eV). At 400 °C, the fraction of the β-NiMoO_4_ phase in the synthesized material increased at the expense of MoO_2_ and α-NiMoO_4_, in addition to a marked increase in the specific surface area (see Table 1 and Figure 2 in Part I of the manuscript [20]), which enhanced the HER performance, as seen in Figure 3a. As reported elsewhere, both MoO_2_ and MoO_3_ and are not active HER electrocatalysts [47]. The highest HER performance was achieved at a calcination temperature of 500 °C, representing an inflection point, beyond which the HER activity decayed rapidly. As shown in Part I of the manuscript [20], by increasing the calcination temperature, the fraction of the β-NiMoO_4_ phase in the material prepared above 500 °C declined, the particle size increased, the pore size distribution shifted to a higher value (due to sintering), and the band gap increased due to enlarged particles.

The best HER performance was observed at the calcination temperature (500 °C) that resulted in the highest amount of β-NiMoO_4_ (~73 wt.%, Table 1 in Part I [20]), indicating that this phase is likely the more active polymorph in the HER, which is also in agreement with the literature [48]. In fact, the relatively better electrocatalytic activity of the β-NiMoO_4_ phase in the HER can be ascribed to its configuration, where the molybdenum ions in its lattice structure are coordinated in a tetrahedral configuration geometry, compared to a pseudo-octahedral configuration geometry present in the α-NiMoO_4_ phase (Figure S4) [26]. In β-NiMoO_4_, the Mo cation is unsaturated, and its empty 4d orbitals are less destabilized than in α-NiMoO_4_. The empty 4d orbitals of Mo in β-NiMoO_4_ are located lower in energy compared to α-NiMoO_4_, as shown by a density-functional theory (DFT) simulation [49]. Since the d-band has been deemed an important activity descriptor in the HER, the localized empty 4d states of Mo near the Fermi levels are available and would enable bonding interactions, proving beneficial to the key step of hydrogen adsorption in the HER [50]. Additionally, by assessing other structural differences between α-NiMoO_4_ and β-NiMoO_4_, it is notable that the major distinction, apart from the coordination Mo, is the distance between the elements in NiMoO_4_ (namely, Ni-Mo and Mo-Mo, while the other bonds (Ni-O and Ni-Ni) are invariable). The increase in the Ni–Ni distance triggers the contraction of the Ni d-band [51], giving a higher density of states near the Fermi level and likely enhancing the kinetics of the Heyrovsky step (Equation (2)) and, in turn, the overall HER performance [15].

The effect of calcination temperature on the HER mechanism appears to be negligible. As shown in Figure 3b, the Tafel slopes of the samples prepared at different calcination temperatures indicate that the HER reaction pathway followed the Volmer–Heyrovsky route even for the materials that exhibited a relatively poor HER activity (900 and 1100 °C). However, the trend of the change in exchange current density is similar to that shown in Figure 3a, with a maximum at 500 °C, indicating that this calcination temperature, indeed, enables the synthesis of the best-performing NiMoO_4_ HER electrocatalyst.

The HER performance of samples synthesized at different calcination times (pre-combusted, as-combusted, 5 min, 20 min, 60 min, 180 min, 360 min, 720 min, and 1440 min) is shown in Figure 3c. Before calcination, the precursor material (labeled as “pre-combusted” in Figure 3c) exhibited a negligible performance in the HER (15 mA cm^−2^). The HER activity improved markedly to 147 mA cm^−2^ with the sample that was combusted (labeled “as-combusted” in Figure 3c) and removed immediately from the furnace after the onset of the combustion reaction. Longer calcination periods enhanced the HER activity, as evidenced by the gradual increase in current density in Figure 3c, ultimately leveling off at 180 min, beyond which extended calcination periods (180, 360, and 720 min) did not influence the HER performance, except at 1440 min where a minor performance decay can be observed.

The calcination time influences an array of physicochemical properties, including the phase composition, band gap, morphology, surface area, and pore volume and size. As it was seen in Section 2.2 in Part I of this study [20], the as-combusted sample exhibited favorable properties such as a high surface area, a semi-developed phase composition, and a comparatively small band gap. The phase composition of this sample consisted of a mix of α-NiMoO_4_, β-NiMoO_4_, NiO, MoO_3_, and MoO_2_ with the latter being the dominant phase (Table 2 in Part I [20]). As the calcination time increased (5 min), MoO_2_ transformed into MoO_3_ in conjunction with an increase in the amount of α-NiMoO_4_ and β-NiMoO_4_ with small changes in the other physicochemical properties (crystallite size, band gap, surface area, pore volume, pore size, and particle size), indicating that the enhanced HER performance seen in Figure 3c was due to the increased presence of NiMoO_4_ and did not stem from the secondary phases. The same observation was noticed in Figure 3a when increasing the calcination temperature from 300 to 400 °C. In Part I of the manuscript [20], it was shown that as the calcination time was prolonged, the samples underwent a small change in the morphology, band gap, surface area, and pore size (Figure 5 in Part I [20]). The phase composition showed a gradual and slow increase in the presence of β-NiMoO_4_ with calcination time (Table 2 in Part I [20]), which coincides with the HER performance trend shown in Figure 3c. This confirms that a better HER activity is concurrent with a high content of β-NiMoO_4_ in the samples. As the calcination time was extended further (720 min), the HER performance varied slightly, during which the phase composition and the other investigated physicochemical properties were invariable (Section 2.2 in Part I [20]). At a prolonged calcination period (1440 min), Figure 3c shows a decline in HER activity, ascribed to a decrease in the β-phase content in the sample, at the expense of an increasing fraction of α-NiMoO_4_. This observation further supports the fact that the β-NiMoO_4_ phase is the more electrocatalytically active polymorph toward the HER. On the other hand, the HER mechanism was not affected by the change in calcination time, as shown in Figure 3d, where the Tafel slope remained constant, while the exchange current density trend (Figure 3d) was similar to that recorded at a constant overpotential in Figure 3c.

#### 2.2.2. Effect of Ni-Mo-Oxide Composition

One of the most important parameters that influences the electrocatalytic activity is the material’s chemical composition. The results presented previously in the text were obtained using NiMoO_4_, with a Ni-to-Mo atomic ratio of 1:1. However, it was interesting to investigate how the composition (relative ratio (atomic) of Ni and Mo in the material) of this electrocatalyst influences its activity in the HER. For this purpose, various compositions of Ni*_x_*Mo_1−*x*_-oxide (*x* = 0.0, 0.2, 0.4, 0.5, 0.6, 0.8, and 1) were produced (Section 2.3 in Part I [20]), and their electrocatalytic activity in the HER is presented in Figure 3e. It is notable that the single-component oxide samples (NiO (*x* = 1) and MoO_3_ (*x* = 0)) exhibited negligible activity in the HER. However, all the binary NiMo-oxide electrocatalysts conveyed a higher HER activity, and a volcano-like trend was observed where the maximum activity occurred at the equiatomic Ni/Mo stoichiometric composition (NiMoO_4_, *x* = 0.5, Figure 3e). The Tafel slopes shown in Figure 3f imply that the HER mechanism of NiO and MoO_3_ is different than those of all the binary compositions. Irrespective of the ratios between Ni and Mo in the binary oxide samples, the prevalent HER mechanism followed the Volmer–Heyrovsky route, while for NiO and MoO_3_, the Volmer step was the RDS. Further, the trend in the exchange current density as a function of Ni*_x_*Mo_1−*x*_-oxide composition is similar to that recorded potentiostatically (Figure 3e), evidencing that the highest HER activity is that of the composition containing the same atomic amount of Ni and Mo (NiMoO_4_), and the trend can be described in the same manner as that related to Figure 3e, as follows.

The increase in HER performance as result of a higher Ni content in the samples (Figure 3e) can be explained by either the change in the specific surface area (extrinsic) or the modification of electronic configuration induced by alloying Ni and Mo. Generally, the electrochemically active surface area (ECSA) is an important parameter that help assess the HER performance of samples with different compositions [52]. However, measuring the ECSA of Ni*_x_*Mo_1−*x*_-oxide samples presented in Figure 3e was not possible since the electrocatalyst layer contained high-surface-area CB, as a conductive additive. Nevertheless, the impact of the ECSA of Ni*_x_*Mo_1−*x*_-oxide samples on their HER activity could be, to a certain extent, assessed by considering the specific (BET) surface area of the pristine Ni*_x_*Mo_1−*x*_-oxide nanostructures, as the same amount of CB was added to all the investigated compositions. 

Since the HER is a heterogeneous reaction occurring at the surface of the electrocatalyst, a smaller particle size would theoretically entail a higher specific surface area, which would result in an improved reaction performance. However, as shown in our previous publication (Figure 7 in Part I [20]), the highest specific surface area (and the smallest particle size) was that of Ni_0.8_Mo_0.2_-oxide (surface area: 110 m^2^/g, particle size: 2–3 nm, respectively), which also exhibited the smallest pore size. This sample, counterintuitively, performed poorly toward the HER, as seen in Figure 3e, indicating that the specific surface area is not the determining factor in the observed HER activity. XRD measurements confirmed that the predominant phase in the best-performing composition (*x* = 0.5, Figure 3e) was β-NiMoO_4_ (Table 4 in Part I [20]), which was previously asserted to be more electrocatalytically active toward the HER than the α-NiMoO_4_ phase. Band gap measurements showed that between *x* = 0.2 and 0.8, the band gap of Ni*_x_*Mo_1−*x*_-oxide hardly changed (Figure 7 in Part I [20]). Additionally, the morphology of the particles did not significantly vary, except for a reduction in the particle size as the atomic fraction of Ni in Ni*_x_*Mo_1−*x*_-oxide increased (Figure S13 in Part I [20]), illustrating that the HER performance in Figure 3e is indeed influenced by the intrinsic properties of the material, one of which is the phase composition of Ni*_x_*Mo_1−*x*_-oxide.

An additional explanation for the volcano trend seen in Figure 3e, exhibiting a maximum at *x* = 0.5, can be supported by theories pertinent to the electrocatalytic activity of pure NiMo alloys in the HER. Namely, it has been established that combining transition-metal elements of different electron configurations results in a synergistic effect, where the performance of the multimetallic alloys is better than its constituent elements. This intrinsic aspect has been discussed exhaustively in the literature, with several theories to explain this phenomenon [15]. Most notably, the “spillover” theory suggests that synergism is due to the surface diffusion of the adsorbed hydrogen between metal elements of a different H_ads_ adsorption affinity. If this theory is applied in the case of Ni*_x_*Mo_1−*x*_-oxide, Ni that exhibits a weaker adsorption strength would serve as a hydrogen source to an adjacent Mo atom which, in turn, acts as a hydrogen trap that facilitates the H_ads_ desorption in the next step (Heyrovsky), leading to enhanced HER kinetics [14]. Another popular theory that discusses synergism is based on the hypo-hyper-d-electronic interactive effect, where an enhancement in the electronic configuration is elicited when alloying transition metals of different d-orbital electron densities, leading to a change in the bonding strength in the resulting bicomponent transition metal-based compound. Irrespective of the causes behind synergism, a phenomenon that likely stems from both electronic and surface considerations, it is reasonable that the best HER performance is achieved when equal amounts of Ni and Mo are used. At the equiatomic composition (*x* = 0.5), both the electronic and surface diffusion effects are expected to be maximized, and if this is applied to Ni*_x_*Mo_1−*x*_-oxide, evaluated in this work, the implication is that these theories, at least partially, support the trend seen in Figure 3e.

#### 2.2.3. Effect of pH

**Figure 4:**
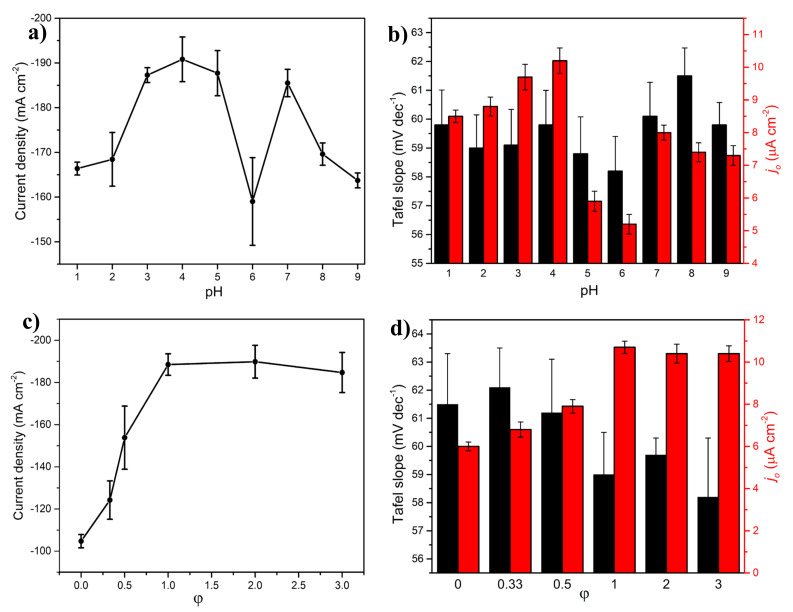
Dependence of the HER current density on the (**a**) pH of the precursor solution and (**c**) Figure 3. ratio (φ). The electrocatalyst layer consisted of NiMoO_4_, carbon black, and Nafion. The current density values were recorded under potentiostatic conditions at an overpotential of −0.58 V after reaching a quasi-steady-state HER rate (1000 s). This was followed by LSV curves at a scan rate of 1 mV s^−1^ and corrected for ohmic (*iR*) drop, shown in Figure S1m,o. The variation in Tafel slope and exchange current density as a function of the (**b**) pH of the precursor solution and (**d**) fuel-to-oxidant ratio (φ); these kinetic parameters were obtained from the Tafel plots presented in Figure S1n,p.

An important parameter in the SCS method is the pH of the precursor solution since it influences a wide range of physicochemical properties in the resulting products and would thereby affect their electrocatalytic performance toward the HER [53]. Assessing the HER performance of the NiMoO_4_ samples prepared at different pH values (Figure 4a) reveal that with a highly acidic precursor solution (pH = 1 and pH = 2), a lower HER activity occurred, after which the performance increased steeply as the pH of the precursor solution approached 4.57 (unadjusted sample), beyond which a small decline in the HER performance was observed at pH = 5. However, a surprising and sharp decrease in activity transpired at pH = 6, which then recovered at pH = 7 and subsequently decreased once again at pH = 8 and pH = 9. A similar trend was observed in Figure 4b, for the exchange current density, while the effect of pH on the HER mechanism of the samples was not evident since all the samples exhibited similar Tafel slopes, all of which point to the prevalence of the Volmer–Heyrovsky reaction route. These results could be explained by the variation in the physicochemical properties of the synthesized samples as a function of pH (the reader should also refer to Section 2.4 in Part I of the manuscript [20]).

In the lower pH region (pH = 1 to pH = 4.57), where HNO_3_ was used to adjust the precursor solution’s pH (originally 4.57), the phase composition of the samples varied slightly, consisting predominantly of β-NiMoO_4_ (Table 5 in Part I [20]); however, the materials’ morphologies were distinctly different. Namely, at pH = 1, the particles were highly agglomerated (Figures S20 and S21 in Part I [20]), and the pore size was large. Additionally, the pore size exhibited a wide distribution consisting of meso- and macropores (Figure 8 in Part I [20]). This led to a lower HER performance (Figure 4a) since the preponderance of highly agglomerated particles can reduce the surface area available for the HER. As the pH in the precursor solution increased from 1 to 4.57, the NiMoO_4_-specific surface area decreased, agglomeration lessened, and the pore size distribution became narrower with a higher presence of the mesopores, leading to an improved HER performance. Upon increasing the pH to 5 by adding ammonia, a change in the phase composition was observed (Table 5 in Part I [20]), as the fraction of α-NiMoO_4_ increased at the expense of β-NiMoO_4_. Additionally, the surface area increased (Figure 8 in Part I [20]) along with the appearance of a small amount of nanorods at pH 5 (Figure S20 in Part I [20]). This led to a decline in the HER performance of the material (Figure 4a) due to the decrease in the β-NiMoO_4_ phase content. At pH = 6, the morphology underwent a drastic increase in the nanorod content (Figures S20 and S21 in Part I [20]) and achieved the highest relative surface area (Figure 8 in Part I [20]) but with the lowest comparative β-NiMoO_4_ content (Table 5 in Part I [20]), resulting in the poorest performance in the HER, as seen in Figure 4a.

At pH = 7, the nanorods were more structurally defined (Figure S20 in Part I [20]), and although the specific surface area was markedly lower than that at pH = 6, the content of β-phase increased incrementally (Table 5 in Part I [20]), while the other investigated physicochemical properties remained unchanged. The reason for the significant HER activity disparity between the samples prepared at pH = 6 and pH = 7 is not clear, but the difference in the aspect ratio of the nanorods could be a controlling factor [54]. As seen in Figure 4a, the HER activity declined again at pH = 9 as this sample exhibited a pronounced degree of agglomeration and a morphology akin to knitting balls (Figure S20 in Part I [20]) with a similar specific surface area (Figure 8 in Part I [20]) and a slightly higher β-phase content as compared to that of pH = 7. It is noteworthy in this context that the sample with the highest surface area and the smallest band gap (pH = 6) achieved the lowest comparative HER performance, as compared to the sample with the lowest BET and the highest band gap (pH 4.57), which displayed the highest comparative HER activity. The origin of this behavior is currently unknown to us. Nevertheless, the effect of pH on the HER mechanism is not evident since all the samples exhibited similar Tafel slopes (Figure 4b), which point to the prevalence of the Volmer–Heyrovsky reaction route. The exchange current density values shown in Figure 4b follow the same trend as that in Figure 4a. The findings here show that both the phase composition and the morphology of the materials are influential factors in predicting the HER performance, similar to what was stated previously in the article.

#### 2.2.4. Effect of Fuel Content

The fuel-to-oxidant ratio (φ) is a prominent figure of merit in the SCS method [55]. In Part I of the manuscript [20], we studied the effect of this ratio on the resulting crystallographic structure, surface morphology, and semiconducting properties of the synthesized materials by varying the amount of the fuel (agar) in the precursor solution (Section 2.5 in Part I [20]). Regarding the HER performance of these samples produced at different φ, Figure 4c shows that the sample that was prepared without using a fuel agent (agar) in the SCS method yielded a current density of ~−105 mA cm^−1^. Upon using a small amount of agar (φ = 1/3), the HER activity increased, and a further increase in φ brought about a significant enhancement in the HER performance until φ = 1, beyond which a plateau region was observed, demonstrating almost a two-fold increase in HER activity compared to the agar-free sample (φ = 0). The HER performance trend shown in Figure 4c agrees with the trend of exchange current density presented in Figure 4d. On the other hand, the variation in the Tafel slopes with samples of different φ seems to be essentially indistinguishable, asserting that the prevalent HER mechanism for the samples prepared with different fuel content is the Volmer–Heyrovsky.

The initial enhancement in HER, seen in Figure 4c, can be attributed to the effect of employing agar, in the SCS procedure, on the physicochemical properties of the synthesized samples. At φ = 0, the XRD patterns show that the sample was predominantly composed of α-NiMoO_4_ (Table 6 in Part I [20]), while the morphology consisted of randomly shaped and sized nanostructures (Part Figures S25 and S26 in Part I [20]) with a narrow pore size distribution, high specific surface area, and small band gap (Figure 9 in Part I [20]). The production of this sample was akin to a coprecipitation method. As the agar content was slightly raised (φ = 1/3), the fraction of the β-NiMoO_4_ phase in the material increased significantly (Table 6 in Part I [20]), the specific surface area decreased, the band gap increased, the pore size distribution remained unchanged (Figure 9 in Part I [20]), and the particles exhibited a more defined shape, with a narrow particle size distribution and a foamy morphology (Figure S25 in Part I [20])—a SCS signature feature. This resulted in improving the HER activity due to morphological transformations and changes in the phase composition, while the negative effects, namely, the reduction in the specific surface area and the increase in the band gap did not seem to play a predominant role in influencing the HER activity of the material. Physicochemical changes in the samples were noticeable until φ = 1, after which the β-NiMoO_4_ content was invariable (Table 6 in Part I [20]), the morphological features slightly changed (Figures S25 and S26 in Part I [20]), and the band gap values stabilized (Figure 9 in Part I [20]). This coincides with the trend achieved with the HER performance (Figure 4c) where the activity leveled off at φ = 1. The variation in the HER performance as a function of φ emphasizes again that the β-NiMoO_4_ content and to a lesser degree the morphology of the material are the most influential parameters in explaining the increase in the HER activity, with no significant influence stemming from the specific surface area, pore volume/size, and/or band gap changes.

### 2.3. Benchmarking and Electrocatalyst Layer Stability

**Figure 5:**
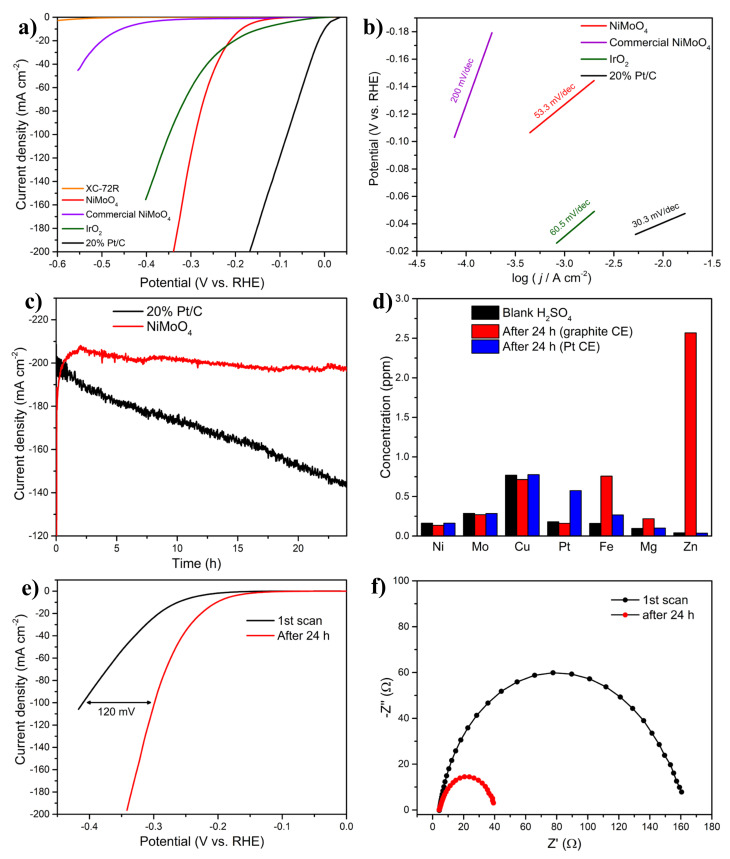
(**a**) Polarization curves of the best-performing NiMo-oxide/CB/Nafion electrocatalyst layer (denoted by NiMoO_4_ and exhibited the following specifications; Nafion (dry weight) loading: 23%, electrocatalyst loading: 0.354 mg cm^−2^, CB loading: 38%, CB type: XC-72R, calcination temperature: 500 °C, calcination time: 6 h, Ni/Mo: 1, precursor solution’s pH: 4.57, φ: 1) and other benchmarks, recorded in 0.5 M H_2_SO_4_ at a scan rate of 1 mV s^−1^ and corrected for ohmic (*iR*) drop (**b**) Tafel regions of the LSV plots in (**a**). (**c**) Long-term stability test of the NiMo-oxide/CB/Nafion electrocatalyst layer (denoted by NiMoO_4_ with the same specifications as those mentioned in (**a**)) and 20 wt.% Pt/C conducted at −0.58 V and −0.33 V, respectively. (**d**) ICP-MS analysis of the blank H_2_SO_4_ electrolyte, and the electrolyte collected after the long-term stability test presented in (**c**) using a graphite rod and a Pt coiled wire as counter electrodes (CE). (**e**) LSV of the electrocatalyst layer before and after the long-term test presented in (**c**). (**f**) Nyquist plot of the electrocatalyst layer before and after the long-term test presented in (**c**), conducted at an overpotential of −0.23 V.

The electrocatalytic activity of the in-house NiMoO_4_ electrocatalyst layer was compared to that of notable benchmarks, as shown in Figure 5a. Commercial platinum supported on CB (20 wt.% Pt/C) has been long utilized as a benchmark material for the HER in PEM systems due to its superior performance. However, its high cost and scarcity impede the proliferation of PEM H_2_ electrolyzers. Additionally, IrO_2_ is known as one of the most catalytically active materials for the oxygen evolution reaction in a PEM electrolyzer, with noteworthy features such as high electrical conductivity and stability in acidic media, in addition to its promising HER performance [56].

As shown in Figure 5a, Pt/C achieved the best HER performance, which is similar to that seen in other works conducted under similar experimental conditions [47]; the outstanding performance of Pt/C is expected due to Pt’s excellent HER kinetics in the acidic media [10]. The in-house synthesized NiMoO_4_/CB/Nafion electrocatalyst layer achieved a good HER activity, exceeding at high overpotentials that of commercial IrO_2_ nanoparticles, albeit still being inferior to Pt/C. It is also interesting that the performance of the in-house electrocatalyst layer was significantly better than that of both NiO and MoO_3_, thereby establishing the presence of a synergistic effect that led to a significant improvement in the electrocatalytic activity, as discussed in Section 2.2.2. Further, the commercial NiMoO_4_ nanostructured sample (Figure 5a) fared very poorly vis-à-vis the (our) in-house-synthesized counterpart, due to the difference in the material morphology—the commercial sample consisted mainly of nanorods, with a drastically different phase composition (absence of the β-NiMoO_4_ polymorph) and a lower specific surface area (Figure S17 in Part I [20]). The HER activity of CB was negligible, indicating that its contribution was limited to imparting better conductivity and stability to the electrocatalyst layer and possibly participating in the reaction mechanism while not being intrinsically electroactive toward the HER. In addition, the use of CB with MoO_3_ and NiO, separately, yielded negligible activity, proving that the synergistic effect seen in Figure 5a is brought about by the cooperative functioning between Ni and Mo in NiMoO_4_, and CB, as mentioned in Section 2.1.2.

The kinetic activities of the samples were also evaluated by measuring their Tafel slopes (Figure 5b). Pt/C exhibited the lowest Tafel slope (33 mV dec^−1^), indicating that the HER on this material is based on the Volmer–Tafel mechanism (Equations (1) and (3)). The in-house electrocatalyst layer, on the other hand, had a Tafel slope of 53.3 mV dec^−1^, which indicates a Volmer–Heyrovsky mechanism (Equations (1) and (2)) with a possible contribution from CB (nonelectrochemical step), as mentioned in Section 2.1.2. IrO_2_ displayed a Tafel slope of 60.5 mV dec^−1^, while the remaining benchmarks lagged significantly behind, indicating high energy barriers for those materials and a rate-limiting step being the initial H^+^ adsorption (Volmer). It is worth mentioning that the substrate’s (glassy carbon) HER performance was negligible, yielding a significant HER overpotential (not shown here) and confirming the absence of any contribution from the substrate to the HER activity of the electrocatalyst layer.

Besides yielding good electrocatalytic properties, an electrocatalyst layer should also be stable, both mechanically and electrochemically. To investigate the electrocatalyst layer’s stability, the long-term electrocatalytic activity of the NiMoO_4_-based electrocatalyst layer was compared to that of the state-of-the-art 20% Pt/C, as portrayed in Figure 5c. The overpotentials used to conduct the long-term stability test were selected (−0.58 V and −0.33 V for NiMoO_4_ and Pt/C, respectively), such that the initial current densities of the samples were similar. Despite the high current density at which the long-term stability test was conducted, the HER performance of the in-house electrocatalyst layer remained stable during 24 h of electrolysis; in fact, its electrocatalytic performance continued to improve with time, evidenced by the increase in the cathodic HER current. In stark contrast, a larger decay in the HER performance was recorded for Pt/C, manifested by the gradual decline in current density (i.e., hydrogen-production activity). The rapid decay in HER current, observed for Pt/C, implies the less-than-optimal electrochemical/structural stability of Pt/C under the experimental conditions employed in this study, as discussed further down. The decay in the HER performance of Pt/C has also been reported elsewhere [57].

The poor electrochemical stability of Pt/C could be due to the well-known electrodeposition phenomena in which trace impurities from the electrolyte solution get deposited on the surface of the electrocatalyst, blocking its active sites [58]. In our study, the impurities could have either originated from the electrolyte or the graphite electrode (counter electrode) in the anodic compartment. ICP-MS analysis (Figure 5d) asserted the presence of trace heavy-ion impurities in the electrolyte (0.5 M H_2_SO_4_) prior to electrolysis. The use of graphite as a counter electrode in the long-term test increased the amount of contaminant ions in the electrolyte after 24 h of electrolysis (Figure 5d), notably Fe, Mg, and Zn, which stemmed from metal impurities present in graphite.

Small amounts of metal ions in the electrolyte solution are sufficient to cover and completely poison the Pt surface, as those foreign elements are much less electrochemically active than Pt [6]. The results herein suggest that when a Pt or Pt-based cathode is used in the HER, a highly pure electrolyte solution is required to avoid a degradation in the electrocatalytic performance. On the other hand, the presence of heavy-metal-ion impurities did not result in any marked deactivation for the in-house NiMoO_4_/CB/Nafion electrocatalyst layer, as the performance exhibited high stability in the long-term experiment (Figure 5c). Those results agree with other studies (IrO_2_ and RuO_2_) showing the low susceptibility of metal oxides electrocatalysts toward fouling in the HER [59]. The use of a Pt counter electrode, however, to circumvent the cleanliness issues of the graphite rod CE, resulted in the preponderance of Pt ions in the solution (Figure 5d), which have a propensity to migrate from the anodic side, under the influence of the electric field, toward the working electrode where they get deposited, leading to a significant increase in the activity of the electrocatalyst layer toward HER (Figure S5), in consonance with findings reported elsewhere [60]. However, it should be noted that the behavior in Figure 5c,e (the increase in current density with time) for the in-house electrocatalyst layer was not a consequence of Pt deposition, as Pt was not used as a counter electrode in this experiment.

**Figure 6:**
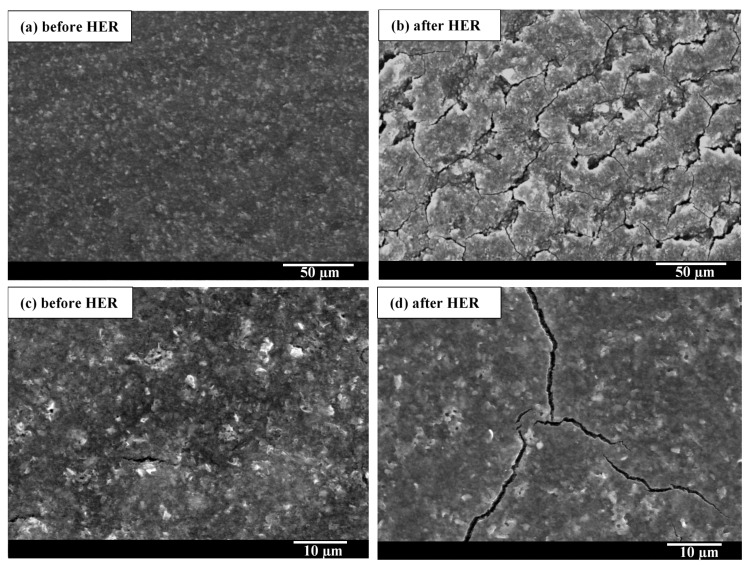
Selected SEM images showing the NiMoO_4_/CB/Nafion electrocatalyst layer (same specifications as those indicated in the caption of Figure 5) before (**a**,**c**) and after electrolysis (**b**,**d**) at different magnifications. The label “Before HER” refers to the sample not subjected to electrolysis, while “After HER” denotes the sample after 24 h of electrolysis.

Figure 5e shows that the performance of the in-house electrocatalyst layer underwent a substantial enhancement in electrocatalytic performance, evident by a two-fold surge in HER current density after 24 h of electrolysis compared to the initially produced (fresh) electrode (the first LSV scan). The EIS portrayed in Figure 5f also displays a marked decrease in the charge-transfer resistance (manifested by a drastic decrease in the semicircle diameter) between the first spectrum and the one recorded after the 24-h electrolysis experiment. To probe the factors that caused the increase in the HER current and the subsequent stability in the performance, the electrocatalyst morphology was recorded before and after long-term electrolysis (Figure 6). Prior to electrolysis, the electrocatalyst layer was smooth with a homogenous distribution of the constituent elements. After the long-term electrolysis test, the surface exhibited a cracked-mud morphology with an apparent increase in roughness, as also shown elsewhere [61]. It is to be noted that the electrode’s immersion in the H_2_SO_4_ electrolyte (without polarization) had no effect on the morphology since SEM images (not shown here) showed that the surface was not affected by mere exposure to the electrolyte. The increase in roughness (seen in Figure 6) was further corroborated by performing profilometry measurements on the samples at different time increments during long-term electrolysis (Figure S6). The mean roughness (Ra) of the sample prior to electrolysis was 0.44 µm, and the value increased to 0.49 µm 15 min after electrolysis and to 0.57 µm after 1 h. At the end of the long-term test (24 h), the roughness factor was determined to be 0.98 µm. Therefore, the rate of the increase in surface roughness was pronounced in the first hour of electrolysis and continued to rise incrementally afterward. The surface cracking and the increase in the electrocatalyst’s layer surface roughness exposed more NiMoO_4_ active sites, which enhanced the HER activity of the electrode, as seen in Figure 5c,e,f [62].

The EDX spectra presented in Figures S7 and S8 show the distribution of the main elements of the electrocatalyst layer before and after the long-term stability test, respectively. Most notably, the amount of oxygen in the sample subjected to long-term electrolysis increased significantly, which can be attributed to the increase in the amount of functional groups on CB due to the extended exposure to H_2_SO_4_, as was shown elsewhere [24,63]. Additionally, it is noteworthy that the ratio of Ni to Mo was preserved after the test. The invariability in the stoichiometric ratio of NiMoO_4_ (Ni/Mo = 1) seen with the EDX results (Figures S7 and S8), combined with the results obtained from ICP-MS (Figure 5d), which showed that there was no increase in Ni or Mo in the electrolyte after the long-term test, suggest that the electrode is durable for HER, under the experimental conditions employed in this study. Additionally, no foreign-ion deposition (Cu, Ti, Zn, and Fe) was detected on the electrocatalyst layer after 24 h of electrolysis, indicating the high fouling resistance of the electrocatalyst, in agreement with the results depicted in Figure 5d. The absence of foreign-ion deposition was also validated by XPS analysis (as will be shown later in the text). The fact that contaminant metal ions did not cover and degrade the HER activity of the electrocatalyst layer throughout the long-term electrolysis test confirms the robustness of NiMoO_4_ and its superior resistance to fouling, unlike Pt/C, which suffered notable degradation in HER performance.

**Figure 7:**
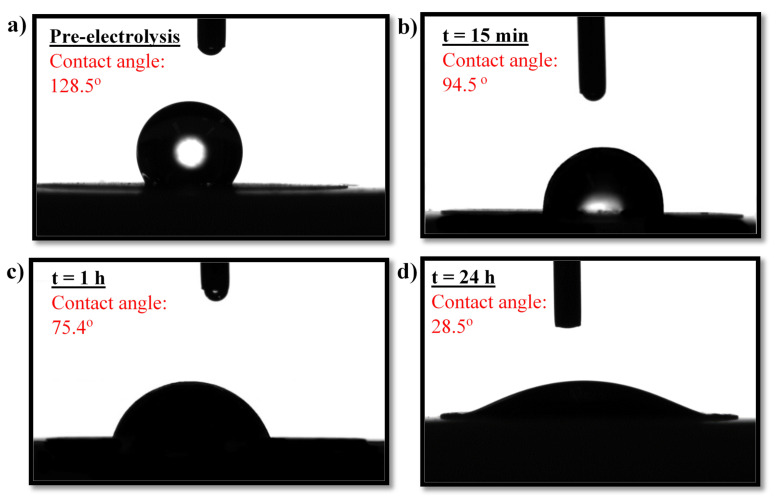
The wettability of the NiMoO4/CB/Nafion electrocatalyst layer (same specifications as Table 5 in Part I). Measured by the contact angle method for different electrolysis durations; (**a**) pre-electrolysis, (**b**) 15 min, (**c**) 1 h, and (**d**) 24 h after electrolysis.

The wettability of the electrode surface could also be a major player in the activity improvement seen during the 24-h electrolysis test (Figure 5c). The contact angle (CA) of the electrocatalyst layer exposed to different polarization periods was measured to survey the change in wettability (Figure 7). The as-prepared material was highly hydrophobic, as evidenced by its high contact angle (126°). After 15 min of electrolysis, the CA of the material declined to 94.5°, signifying an increase in wettability. This effect was further exasperated with an increase in the electrolysis time, where the contact angle further decreased to 75.4° after 1 h. After 24 h of electrolysis, the electrocatalyst’s surface became highly hydrophilic (CA = 28.5°), indicating that long-term electrolysis led to a drastic wettability enhancement in the material. The enhanced wettability brings about a better interaction between the electrocatalyst and the electrolyte, efficient bubble detachment (the H_2_ bubbles generated during the electrocatalytic process can pin on the surface of the electrode and are difficult to release when the surface is hydrophobic [64]), in addition to an improved effective surface area (the area accessible to the electrolyte solution that can participate in the HER) [61].

The increase in wettability may be ascribed to several reasons. Nafion contains both a hydrophobic backbone (tetrafluoroethylene) and hydrophilic side chains (sulfonic acid) in its chemical structure. In the dry mode, Nafion is hydrophobic, but when exposed to water, its hydrophobic nature prevails due to the water-absorbing affinity of the ionomers. Water is absorbed into the hydrophilic domains, solvating the acid groups and causing the polymer to swell [65], leading to a decreased CA, i.e., better wettability. A previous study has shown that before being placed in contact with water, the CA of different types of Nafion membranes ranges between 90–110° [66]. However, with extended exposure to water, the contact angle drops by ~15–20°, leveling off after ~2000 s. Since the reduction in CA seen in Figure 7 was markedly more pronounced than what would be expected from mere Nafion swelling, other factors must be considered. As mentioned previously, CB contains ample functional groups and is generally hydrophobic [67]. The generation of oxygen-containing functional groups due to the exposure of the electrocatalyst to H_2_SO_4_ [68] and surface hydroxylation [62] can also enhance the hydrophilicity and consequently the surface area accessible to the aqueous electrolyte [24]. Surface deformation (roughening), evident from the images in Figure 6, could also be a contributing factor to the rise in wettability. Increased wettability stemming primarily from Nafion swelling would be compounded by the increased surface roughness of the electrocatalyst layer with long-term electrolysis [69].

From the results above and other findings in the literature, it can be conjectured that the increase in the electrocatalytic performance (seen in Figure 5c,e,f) cannot be limited to only one factor. Part of this effect stems from the increase in the wettability of the oxide surface as a result of the electrolyte spreading over regions made hydrophilic by the reductive treatment. Another possible factor is the deformation of the electrocatalyst’s surface due to the vigorous production of H_2_. Other aspects discussed elsewhere that could also explain the performance improvement include the increase in the volume of the unit cell [70] and H-chemisorption on the electrode surface, which could contribute to the formation of favorable active sites [61]. Additionally, changes in surface chemistry could be a factor, which is discussed in the next section.

**Figure 8:**
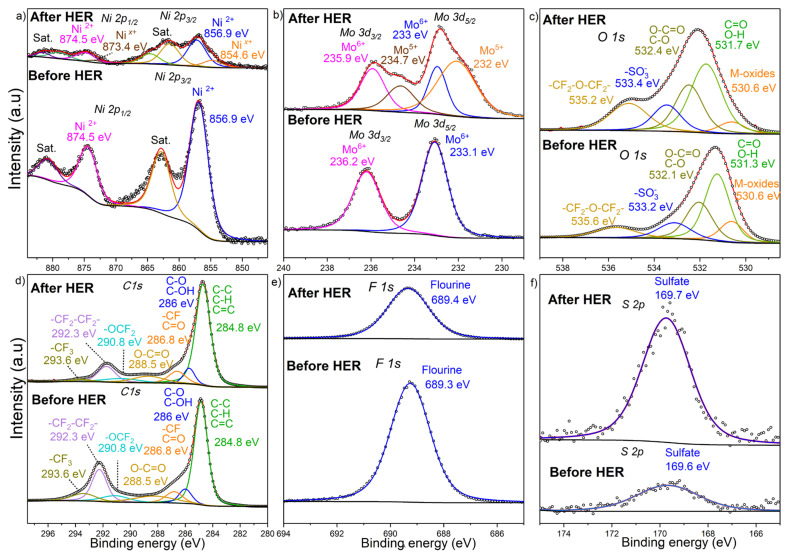
XPS analysis of the NiMoO_4_/CB/Nafion electrocatalyst layer (same specifications as those indicated in the caption of Figure 5) conducted before and after 24 h of electrolysis, performed at an overpotential of −0.58 V, portraying the elemental scans of the electrocatalytic constituents: (**a**) Ni 2p, (**b**) Mo 3d, (**c**) O 1s, (**d**) C 1s, (**e**) F 1s, and (**f**) S 2p. The label “Before HER” refers to the XPS spectrum of the sample before electrolysis, while “After HER” denotes the XPS spectrum of the sample after the 24-h electrolysis test. The points represent the experimental data, while the lines represent the deconvoluted spectra.

The variation in the surface chemistry of the electrocatalyst layer following the 24 h electrolysis test was studied by XPS (Figure 8). The signals obtained in the XPS spectra can be traced to three major components that constitute the electrocatalyst layer: (1) electrocatalytic material NiMoO_4_ (Ni, Mo, and O), (2) CB (C and O), and (3) Nafion (C, O, F, and S). The high-resolution spectrum of Ni (Figure 8a), before electrolysis, shows a typical spin–orbit doublet corresponding to Ni 2p_3/2_ and Ni 2p_1/2_ and their satellite peaks. The deconvolution of the spectrum revealed two main peaks at 856.9 and 874.5 eV, separated by an energy gap of 17.6 eV, indicating the predominant presence of Ni^2+^ [71]. Long-term electrolysis influenced the valency state of Ni since the spectrum obtained after the long-term test (Figure 8a) indicates the presence of an additional oxidation state at a lower binding energy (Ni*^x^*^+^, 854.6 eV and 873.4) than that of Ni^2+^.

The high-resolution Mo spectrum (Figure 8b) demonstrates that before electrolysis, the sample exhibited a typical spin–orbit doublet corresponding to Mo 3d_5/2_ and Mo 3d_3/2_ with binding energies of 233.1 and 236.2 eV, respectively, evidencing the prevalence of the Mo^6+^ oxidation state [72]. After the 24 h electrolysis test, the Mo core-level spectrum showed a new spin–orbit doublet (232 eV and 234.7 eV) corresponding to Mo^5+^ [73]. Considering the formation of lower-valency Ni species (Figure 8a), the valency changes in Ni and Mo could be attributed to the formation of lower-oxidation Ni-Mo-oxide intermediates compared to the initial (freshly synthesized) NiMoO_4_.

The O 1s core-level spectrum (Figure 8c, before electrolysis) displays several peaks, among which the one at 530.6 eV corresponds to the metal-oxide component in NiMoO_4_ [71], while the peaks at 531.3 eV and 532.1 eV stem from oxygen-rich CB surface functional groups (carbonyl, carboxylate, ether, quinine, etc.) and adsorbed water molecules [41]. The peak at 533.2 eV is related to the sulfonic acid side group (–SO_3_^−^) found in Nafion, while the one at 536.5 eV is consistent with the oxygen contained in ether groups (–F_2_C–O–CF_2_–) of Nafion [74]. After the 24 h stability test, the discernible changes are those related to the increase in the peak intensity of O–C=O (532.4 eV), which suggests an increase in oxygen-rich functional groups after electrolysis and agrees with the premise put forward previously in the text about the role these functional groups play in increasing the wettability of the electrocatalyst layer (Figure 7). Additionally, the intensity of the peak corresponding to –SO_3_^−^ (533.4 eV) appears to have increased after the long-term test due to the presence of residual H_2_SO_4_ on the electrocatalyst layer; alternatively, this increase could be ascribed to the reorganization of Nafion caused by the electrocatalyst layer’s long exposure to H_2_SO_4_, resulting in a higher amount of sulfur functional groups on the surface [75]. Nafion reorganization has been explained in previous studies based on the rotation of the Nafion polymer chain upon contact with H_2_SO_4_, which directs the sulfonic functional groups of Nafion to the surface [75]. Another notable intensity increase is associated with the fluoroether-related peak at 535.2 eV.

Carbon (Figure 8d, before electrolysis) shows a convoluted spectrum, which consists of multiple peaks. Upon deconvolution, the main peaks are those of adventitious carbon, C–H, C=C, and C–C at 284.8 eV [76]; –C–O and C–OH at 286 eV arising from CB functional groups and the adsorbance of water or hydroxyl groups on the electrocatalyst layer; –C–F and C=O at 286.8 eV originating from Nafion and CB, respectively [77], and O–C=O at 288.5 eV ascribed to the functional groups found on the surface of CB. The peak at 290.8 eV is consistent with –OCF_2_, while the high intensity peak seen at 292.3 eV refers to –CF_2_–CF_2_; the former and latter are species present in the molecular structure of Nafion [74]. The shoulder at 293.6 eV is ascribed to –CF_3_ species. Some contribution can also come from a typically broad and low-intensity peak at ~291 eV, corresponding to the π → π* plasmon excitation (shake-up satellites of unsaturated species) [41]. After electrolysis, the C 1s spectrum was manifested by an intensity reduction specific to–OCF_2_ (290.8 eV), –CF_2_–CF_2_– (292.3 eV), and –CF_3_ (293.6 eV) due to Nafion’s reorganization [75]. Additionally, a slight increase in the peaks specific to O–C=O (288.5 eV) and C–O, C–OH (286 eV) was observed, which agrees with what was asserted previously about the increase in the oxygen-based functional groups when CB is exposed to H_2_SO_4_ (Section 2.3). The XPS spectra (and interpretation) of the pristine CBs, used in this study, are included in Figure S3.

The core-level spectra of fluorine (F 1s) shown in Figure 8e (before electrolysis) display a peak at 689.3 eV corresponding to the fluorine element in Nafion, the intensity of which increased following electrolysis due to the reorganization of the Nafion layer [75].

The core-level spectra of sulfur (Figure 8f) consist of one peak at 169.6 eV corresponding to –SO_3_^−^, which encountered an increase in intensity during long-term electrolysis due to the exposure to H_2_SO_4_ and Nafion rearrangement. 

It is important to mention that XPS analysis of heavy-ion metals such as Cu, Fe, and Zn revealed the absence of those elements on the surface of the electrocatalyst after the long-term electrolysis, confirming the excellent fouling resistance of NiMoO_4_.

In view of the information gleaned from the characterization techniques following the long-term electrolysis test, it can be noted that a combination of factors contributed to the progressive increase in the HER activity of the NiMoO_4_-based electrocatalyst layer observed in Figure 5c. The change in surface chemistry seen in XPS could be an influential factor in the performance enhancement. Lower-valence-state metals could exhibit a higher intrinsic conductivity that would boost the electrocatalytic performance toward the HER. Additionally, the other previously discussed factors (wettability and roughness) prove important in this phenomenon due to their role in allowing higher ion accessibility to the active sites, embedded in the electrocatalyst, and increasing the active surface area participating in the HER.

Finally, it is important to discuss the price aspect of the catalysts as the commercial attractiveness of the current PEM electrolyzers suffers from low commercial interest due, in part, to their expensive nature stemming from the use of PGM. The price and availability of the catalysts are major factors to address when seeking a material that could potentially replace Pt. To assess the economic viability of the tested benchmark metals compared to the in-house synthesized NiMoO_4_ electrocatalytic material, we considered their current market prices (Figure S9a). To get a representative metric, the normalized cost/HER activity (USD/mA) ratio was computed, as shown in Figure S9b. For NiMoO_4_, the cost to yield 1 mA in HER was ~0.02 cents compared to ~15 cents for Pt and a staggering ~1.8 dollars for IrO_2_. The earth-abundant and low-price nature of NiMoO_4_ compared to the precious noble metals in addition to the high electrochemical and mechanical stability of NiMoO_4_ (Section 2.3) demonstrate that NiMoO_4_ is an economically sound candidate with a good HER activity and electrochemical stability, making it a potentially viable material for the HER in the acidic media.

## 3. Materials and Methods

### 3.1. Sample Preparation

Various NiMoO_4_ nanostructures, prepared at different conditions, were synthesized according to the procedure outlined in our previous study [19]. Briefly, a solution combustion synthesis method was employed in which agar was used as fuel, Ni(NO_3_)_2_ as an oxidant and Ni ion source, while (NH_4_)_6_Mo_7_O_24_ served as a source of Mo cations. A solution containing the fuel and ion precursors was prepared and heated up to 95 °C to solubilize agar. After leaving the solution to cool at ambient conditions, its water content was then removed in a convection oven to get a dried precursor material. A self-propagating combustion reaction was initiated upon placing the precursor material in a preheated furnace at a specific temperature, yielding a fluffy and voluminous powder within seconds. A systematic and comprehensive study discussing the variation in the physicochemical properties of NiMoO_4_ nanostructures with different experimental conditions (calcination temperature, calcination time, Ni/Mo atomic ratio, precursor solution’s pH, and fuel-to-oxidant ratio (φ)) was presented in Part I of this study [20]. Unless otherwise stated, the calcination temperature and time were 500 °C and 6 h, respectively, the pH of the precursor solution was 4.57, and both the Ni/Mo and fuel-to-oxidant ratios (the fuel-to-oxidant ratio is denoted by the equivalence ratio (φ)) were 1. pH changes were made using diluted HNO_3_ (Fisher Scientific, Hampton, NH, USA, 68–70% purity) and NH_4_OH (Fisher Scientific, Canada, 28–30% purity).

### 3.2. Electrochemical Characterization

Electrochemical measurements were conducted in 0.5 M H_2_SO_4_ (Fisher Scientific, Hampton, NH, USA, 98% purity) using a three-electrode electrochemical cell connected to an Autolab potentiostat (PGSTAT30, Metrohm, Mississauga, ON, Canada). The working electrode consisted of a glassy carbon electrode substrate (GCE, surface area: 0.283 cm^2^)) covered with a thin layer of the electrocatalytic material investigated in this work, while a platinum coiled wire and a saturated calomel electrode (SCE) were used as the counter and reference electrodes, respectively. However, all the potentials reported in this study were converted to a reversible hydrogen electrode (RHE) according to the following equation: E (RHE) = E (SCE) + 0.244 + 0.0591pH.

The electrocatalyst layer was prepared by making a suspension containing the actual electrocatalyst, NiMo-oxide powder, and carbon black in water and propanol (80:20 volume). Subsequently, Nafion (St. Louis, MO, USA, 5 wt.%) was added to the suspension and served as a binder and cation (proton) exchange ionomer. Unless otherwise stated, the amount of Nafion added to the mixture was 23 wt.% (dry weight), the electrocatalyst (NiMo-oxide) loading was 0.283 mg cm^−2^, and the NiMo-oxide/CB mixture consisted of 38 wt.% of CB (the amounts of the NiMo-oxide powder, CB, and Nafion were varied to study their effect on the HER performance). The mixture was then sonicated in an ice-filled bath sonicator for 30 min to ensure adequate dispersion and homogeneity of the mixture. A 20 µL aliquot was drawn directly from the sonicated suspension and dropped on the mirror-polished GCE and subsequently dried at room temperature in an argon atmosphere. Prior to using the GCE, its surface was cleaned with reverse-osmosis (RO) water and polished with 0.05 µm alumina suspension (Anamet, Montreal, QC, Canada) on a porous neoprene cloth (Black Star, Anamet, Montreal, QC, Canada) until a clean and mirror-like surface was obtained. The GCE was then washed with RO water to remove any alumina residuals and subsequently wetted with ethanol and dried with argon. We noticed that using ethanol rather than RO in the last washing step significantly facilitated the drop-casting step and led to a more homogenous coverage on the GC (Figures S2a and S10).

Prior to the electrochemical measurements, the electrolyte was purged with argon (MEGS Specialty Gases Inc., Montreal, QC, Canada, 99.998 wt.% purity) for 30 min to remove any dissolved oxygen and carbon dioxide. To ensure a reproducible starting surface condition for all the investigated electrocatalysts and to also stabilize the electrode surface, the working electrode was first potentiostatically polarized for 1000 s at −0.58 V vs. RHE (−0.85 V vs. SCE), under stirring (650 RPM, stirring bar: 4 mm × 35 mm). The stirring step was vital to disperse the H_2_ bubbles formed on and attached to the electrode surface. Subsequently, linear sweep voltammetry (LSV) was performed in the potential range from −0.58 to 0.07 V at a scan rate of 1 mV s^−1^. The LSVs presented in this work were smoothed out for noise elimination (which originated from the attachment of H_2_ bubbles on the electrode surface) and better visualization of the results. Commercial platinum (20 wt.% Pt/C, Fisher Scientific, Hampton, NH, USA), IrO_2_ (Fisher Scientific, Hampton, NH, USA), and NiMoO_4_ (Alfa Aesar, Tewksbury, MA, USA) powders were also assessed for comparative purposes under the same conditions (control electrocatalysts).

Electrode-stability tests were conducted for 24 h in 0.5 M H_2_SO_4_ for the in-house synthesized NiMoO_4_ and commercial 20% wt.% at −0.58 V and −0.33 V, respectively. A graphite rod was used as a counter electrode in the long-term stability test instead of Pt; this is to avoid the dissolution and crossover of platinum ions from the anodic to the cathodic compartment and their electrodeposition on the electrocatalyst, which would interfere with the observed HER performance. The graphite rod was separated from the main cell compartment by a glass frit to prevent oxygen formed on it to reach the cathode and avoid impacting the HER activity.

Nyquist plots were recorded using electrochemical impedance spectroscopy (EIS) measurements performed in 0.5 M H_2_SO_4_ at −0.23 V over a frequency range from 100 kHz to 0.1 Hz, with an AC amplitude of ±10 mV.

### 3.3. Characterization Techniques

N_2_ adsorption/desorption measurements (−196 °C) were conducted using a Micromeritics TriStar 3000 instrument (Micromeritics, Norcross, GA, USA) to determine the specific surface area and pore volume of the samples. Before the measurements, the samples were degassed for 12 h at 120 °C. The Brunauer–Emmett–Teller model (BET) was applied to obtain the specific surface area. The pore volume was determined using the adsorption and desorption branches of the isotherms.

Band gap measurements were carried out with a ThermoScientific Evolution 300 instrument (Thermo Fisher Scientific, Waltham, MA, USA), utilizing UV/Vis spectrometry with a 266 nm excitation laser. KBr was used to obtain a background spectrum and as a diluent for the studied NiMo-oxide samples using a KBr-to-sample ratio of 90:10 wt.%. X-ray diffraction (XRD) was employed using a Bruker D8 discovery X-ray diffractometer Bruker, Billerica, MA, USA) with a two-dimensional Vantec-500 detector and CuK (λ = 1.54056 Å). The morphology of the electrocatalyst was studied using field emission scanning electron microscopy (FE-SEM, Hitachi S-4800, Tokyo, Japan). Raman spectra were obtained by a DXR2 Raman spectrometer (Thermo Fisher Scientific, Waltham, MA, USA) to study the graphitization degree of CB, using a 532 nm laser source. Inductively coupled plasma optical emission spectroscopy (ICP-MS, iCAP 6500 dual view, Thermo Scientific, Waltham, MA, USA) was used to analyze the elements present in the electrolyte solution before and after the long-term electrolysis test. Profilometry measurements were conducted using a DektakXT stylus (Bruker, Billerica, MA, USA) to obtain the thickness of the electrocatalyst layer, a topographical profile of the electrocatalyst, and its roughness average (Ra), at different electrolysis time periods. A goniometer (OCA 15EC) equipped with a CCD camera (Data Physics, Filderstadt, Germany) was used to measure the contact angle of the samples at different electrolysis time intervals. X-ray photoelectron spectroscopy (XPS) measurements were performed to reveal the surface chemistry and elemental distribution of the electrocatalyst using a ThermoScientific K-Alpha spectrometer (Waltham, MA, USA) with non-monochromatic source of Al Kα (1486.6 eV photon energy). Prior to analysis, the samples were placed in a highly vacuumed desiccator to remove any moisture and humidity.

## 4. Conclusions

NiMo-oxide nanostructures, produced and characterized in our previous parametric study (Part I of this study [20]), were investigated as electrocatalysts for the hydrogen evolution reaction in the acidic media. The effect of various experimental (synthesis and non-synthesis) conditions was explored (the effects of Nafion loading, electrocatalyst loading, carbon black loading, carbon black grade, calcination temperature, calcination time, Ni/Mo atomic ratio, precursor solution’s pH, and fuel-to-oxidant ratio).

The change in the Nafion loading in the electrocatalyst layer influenced the HER performance. At low loadings, the catalytic activity was limited by poor ionic conductivity. At high loadings, mass-transfer and electrical conductivity limitations became prevalent, leading to a decline in the HER activity. The variation in the catalyst (NiMoO_4_) loading resulted in a noticeable increase in the HER performance at low loadings, after which a plateau region was achieved at higher loadings. CB was used to increase the performance of the electrocatalyst layer by enhancing its electrical conductivity. The HER activity of the electrocatalyst layer saw a significant increase at low CB loadings, leveling off with further CB addition. The effect of CB grade was also investigated, showing that at high loadings, all CBs gave the same performance, while at low loadings, a marked difference in HER activity was observed among the studied CB types.

The dependence of the HER activity on the NiMoO_4_ calcination temperature resembled a volcano-like plot where a temperature of 500 °C achieved the best HER activity. The influence of the calcination temperature on the HER could be explained by the change in phase composition with the temperature, as the amount of the more HER electrocatalytically active β-phase was maximized at 500 °C, while the other polymorph, α-phase, and secondary phases (NiO and MoO_3_) were manifested at the other investigated calcination temperatures leading to a lower HER performance. The influence of calcination time did not yield a strong influence on the HER activity. At short calcination periods, NiMoO_4_ samples contained a considerable content of secondary phases, which subsided with longer calcination periods, concomitant with an increasing amount of β-phase in the material, thereby yielding a higher HER performance. Studying the influence of the Ni/Mo atomic ratio on the HER electrocatalytic activity revealed the stoichiometric sample (Ni/Mo = 1, i.e., NiMoO_4_) exhibited the best performance. Regarding the effect of the precursor solution’s pH, two distinctive regions were visible; for the NiMoO_4_ samples prepared in the acidic media, the HER performance fared better than those produced in the alkaline media. In the acidic media, the samples were less agglomerated with a high amount of β-phase, while in the alkaline solution, the morphology of the materials changed to nanorods and agglomerated microspheres with a lower β-phase content. The effect of the fuel-to-oxidant ratio showed that the samples prepared with a relatively lower amount of fuel (agar) exhibited a comparatively lower activity in the HER; however, the activity leveled off beyond φ = 1 (in the fuel-rich region), which coincided with the fact that the amount of β-NiMoO_4_ was invariable in the fuel-rich region.

Long-term electrolysis was conducted on the best-performing NiMoO_4_ electrocatalyst layer, obtained from the parametric study, yielding a stable behavior over 24 h compared to the state-of-the-art Pt/C material, which underwent a substantial degradation in HER activity. It was also observed that the performance of the in-house electrocatalyst improved in the first few hours of electrolysis and then remained almost stable compared to that of Pt/C which degraded rapidly over the course of the stability test. Profilometry measurements revealed that the surface’s wettability increased with time, which resulted in better accessibility of the electrolyte to the active materials of the electrocatalyst layer. SEM images showed an increased surface roughness, which contributed to an enhancement in the electrocatalytic performance, while EDX analysis asserted that the surface was immune to the electrodeposition of heavy-metal ions from the electrolyte, establishing its robustness. XPS analysis indicated that the surface chemistry of the electrocatalyst changed after the long-term stability test, which helped interpret the observed enhancement in HER performance.

## Figures and Tables

**Table 1:** The effect of CB loading on the surface area and pore volume of NiMoO_4_/CB mixtures.

Material	BET Surface Area (m^2^/g)	BJH Pore Volume (cm^3^/g)
NiMoO_4_	25.8	0.130
XC-72R	238	0.560
91% NiMoO_4_ + 9% XC-72R	61.6	0.182
71% NiMoO_4_ + 29% XC-72R	112.7	0.262

**Table 3:** Elemental composition of the pristine CBs, obtained through XPS quantitative analysis.

Sample		Element (at.%)	
	Carbon	Oxygen	Sulfur
XC-72R	99.48	0.33	0.18
Black Pearls 2000	97.01	2.65	0.34
LITX 2000	94.89	4.09	0.18
PBX 51	98.64	1.36	*
XCmax 22	97.24	2.59	0.17

* Not detected.

## Data Availability

Data are available from the authors upon request.

## Supplementary Materials

The following supporting information can be downloaded online. Figure S1: LSV and Tafel plots of the effect of (a,b) Nafion loading, (c,d) electrocatalyst (NiMoO_4_) loading, (e,f) carbon black loading, (g,h) calcination temperature, (i,j) calcination time, (k,l) Ni/Mo atomic ratio, (m,n) precursor solution pH, and (o,p) the fuel-to-oxidant ratio. Measurements were conducted in 0.5 M H_2_SO_4_ at a scan rate of 1 mV s^−1^ and corrected for ohmic (*iR*) drop. The electrocatalyst layer consisted of NiMoO_4_ and carbon black (XC-72R) and Nafion. Figure S2: (a) A side-view representation of the NiMoO_4_/CB/Nafion electrocatalyst layer portraying its thickness; (b) a topography profile used to obtain the thickness of the catalytic layer. Both methods converged with the goniometer and profilometer yielding 32 µm and 33 µm, respectively. Figure S3: XPS survey and high-resolution C 1s and O 1s spectra of different types of carbon black (XC-72R, Black Pearls 2000, LITX 200, PBX 51, and Xcmax 22). Figure S4: Partial view representation of α-NiMoO_4_ and β-NiMoO_4_ polymorphs. Figure S5: Long-term HER (water electrolysis) test employing the NiMoO_4_/CB/Nafion layer as a working electrode (cathode) and a Pt coil as a counter electrode. The performance was clearly ramping as the experiment progressed due to the dissolution of Pt and the migration of its ions to the cathodic side, where they get electrodeposited on the working electrode improving its electrocatalytic HER performance. Figure S6: Profilometry measurements of the NiMoO_4_/CB/Nafion electrocatalytic layer (a) before and after (b) 15 min, (c) 1 h, and (d) 24 h of water electrolysis in 0.5 M H_2_SO_4_ at an overpotential of −0.58 V. Figure S7: EDX analysis of the NiMoO_4_/CB/Nafion electrocatalyst layer before the 24-h electrolysis test in 0.5 M H_2_SO_4_ at an overpotential of −0.58 V showing the (a) SEM images over which the analysis was conducted, (b) elemental composition of the elements of interest, and (c) their respective mapping distribution. Figure S8: EDX analysis of the NiMoO_4_/CB/Nafion electrocatalyst layer after the 24-h electrolysis test in 0.5 M H_2_SO_4_ at an overpotential of −0.58 V, showing the (a) SEM images over which the analysis was conducted, (b) elemental composition of the elements of interest, and (c) their respective mapping distribution. Figure S9: (a) The current market price of Ir, Pt, Ni, and Mo in USD; (b) the cost-normalized HER performance of the material. Figure S10: An optical-microscopy representation of the NiMoO_4_/CB/Nafion electrocatalyst layer (magnification 10×).

## Sample Availability

Samples of the compounds are not available from the authors.
