# Supplementary material for: Part II: NiMoO4 Nanostructures Synthesized by the Solution Combustion Method: A Parametric Study on the Influence of Material Synthesis and Electrode-Fabrication Parameters on the Electrocatalytic Activity in the Hydrogen Evolution Reaction
Source: Molecules. 2022 Feb 10;27(4):1199. doi: 10.3390/molecules27041199 (PMC8876296; doi:10.3390/molecules27041199)
Supplement: Supplementary file 1 [file molecules-27-01199-s001.zip › molecules-1525710-supplementary.pdf]

Supplementary Information

Part II: $NiMoO_4$ Nanostructures Synthesized by the Solution Combustion Method: A Parametric Study on the Influence of Material Synthesis and Electrode-Fabrication Parameters on the Electrocatalytic Activity in the Hydrogen Evolution Reaction

Mahmoud Bassam Rammal*, Vincent El-Ghoubaira and Sasha Omanovic

Department of Chemical Engineering, McGill University, 3610 University Street, Montreal, QC, H3A 0C5, Canada

*Correspondence: mahmoud.rammal@mail.mcgill.ca

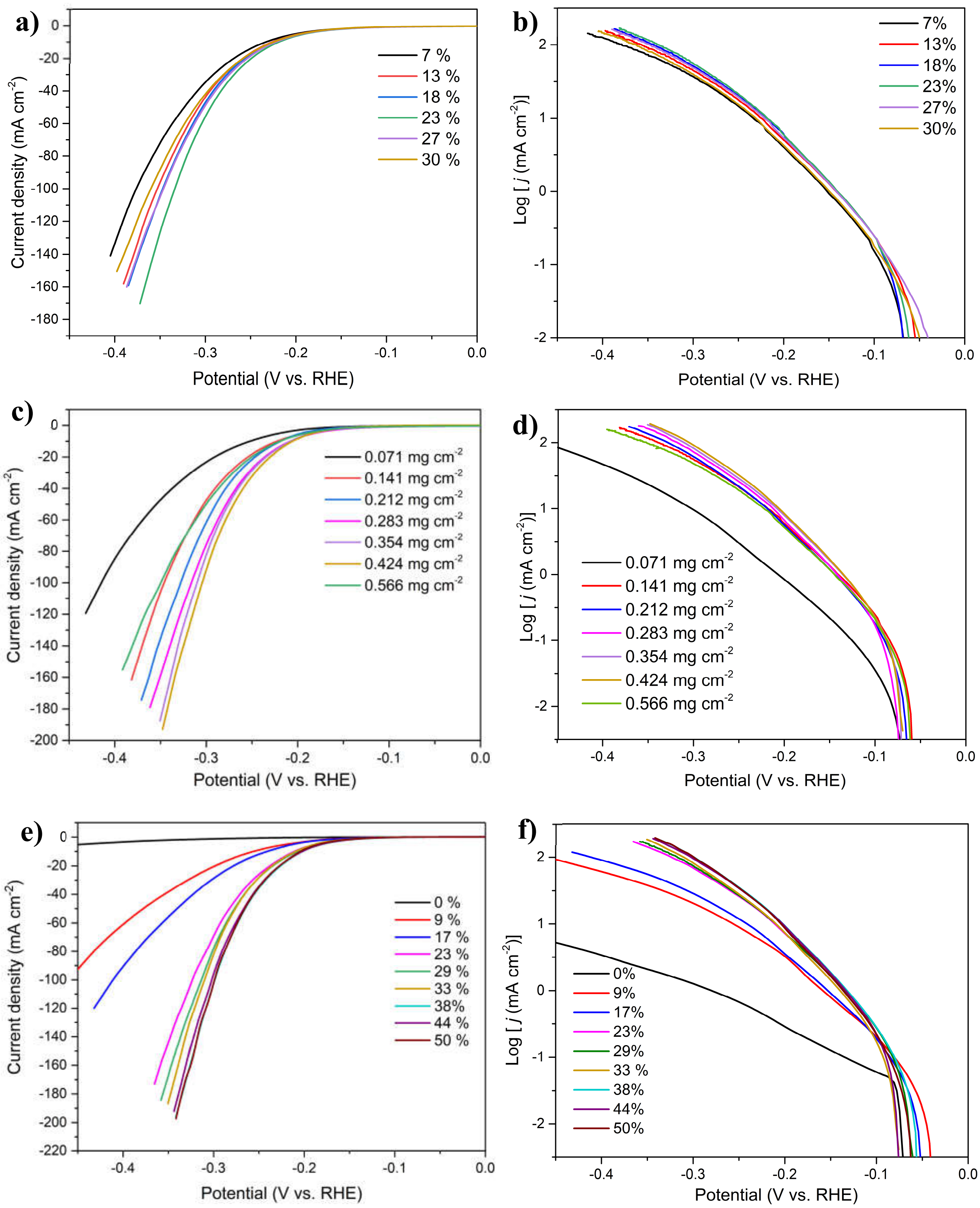
a)
Current density (mA cm-2)
Potential (V vs. RHE)
7 %
13 %
18 %
23 %
27 %
30 %
b)
Log [j (mA cm-2)]
Potential (V vs. RHE)
7%
13%
18%
23%
27%
30%
c)
Current density (mA cm-2)
Potential (V vs. RHE)
0.071 mg cm-2
0.141 mg cm-2
0.212 mg cm-2
0.283 mg cm-2
0.354 mg cm-2
0.424 mg cm-2
0.566 mg cm-2
d)
Log [j (mA cm-2)]
Potential (V vs. RHE)
0.071 mg cm-2
0.141 mg cm-2
0.212 mg cm-2
0.283 mg cm-2
0.354 mg cm-2
0.424 mg cm-2
0.566 mg cm-2
e)
Current density (mA cm-2)
Potential (V vs. RHE)
0 %
9 %
17 %
23 %
29 %
33 %
38%
44 %
50 %
f)
Log [j (mA cm-2)]
Potential (V vs. RHE)
0%
9%
17%
23%
29%
33 %
38%
44%
50%

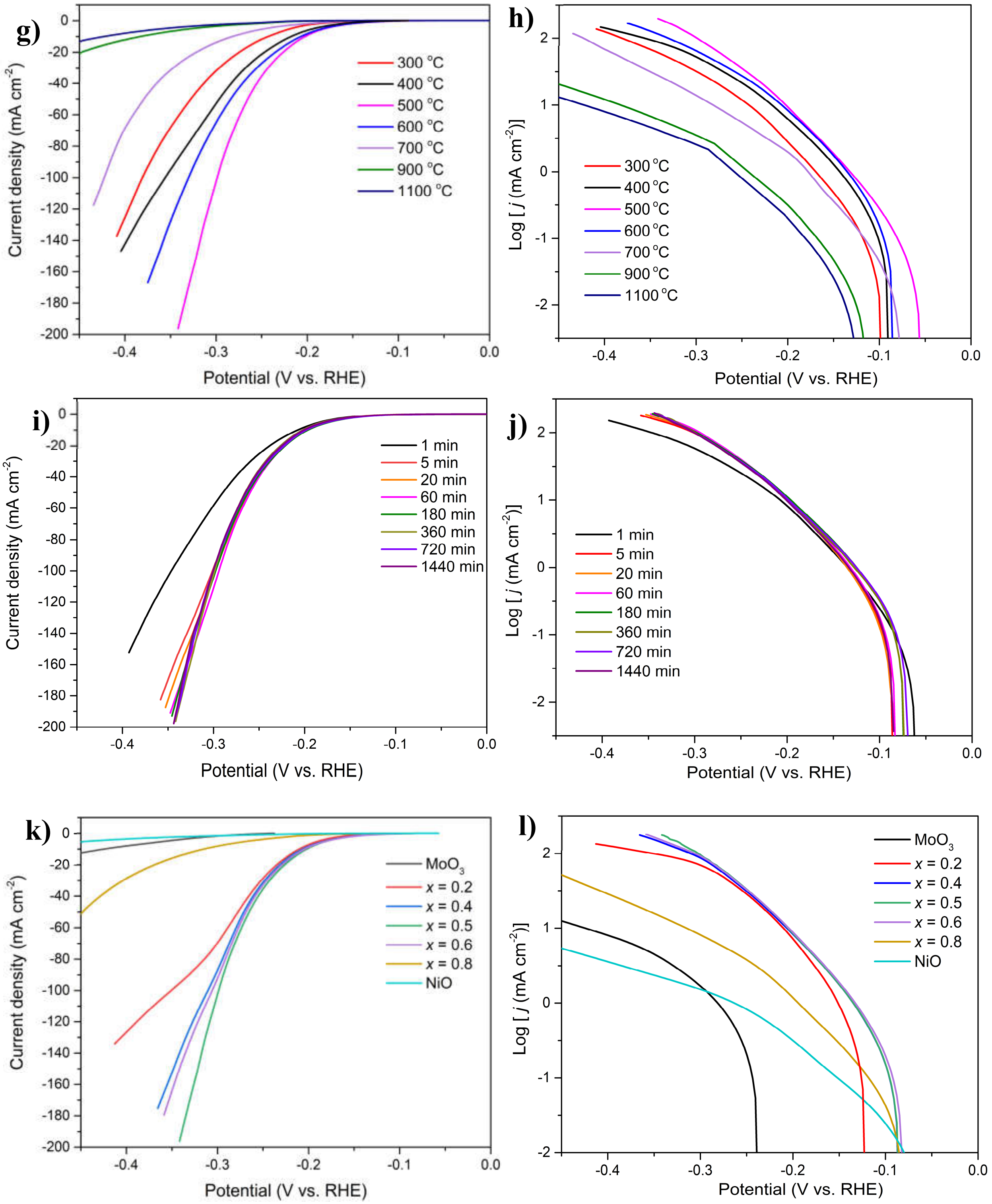

g)
h)
i)
j)
k)
l)
Current density (mA cm^{-2})
Log [j (mA cm^{-2})]
Potential (V vs. RHE)
300 °C
400 °C
500 °C
600 °C
700 °C
900 °C
1100 °C
1 min
5 min
20 min
60 min
180 min
360 min
720 min
1440 min
MoO_3
x = 0.2
x = 0.4
x = 0.5
x = 0.6
x = 0.8
NiO

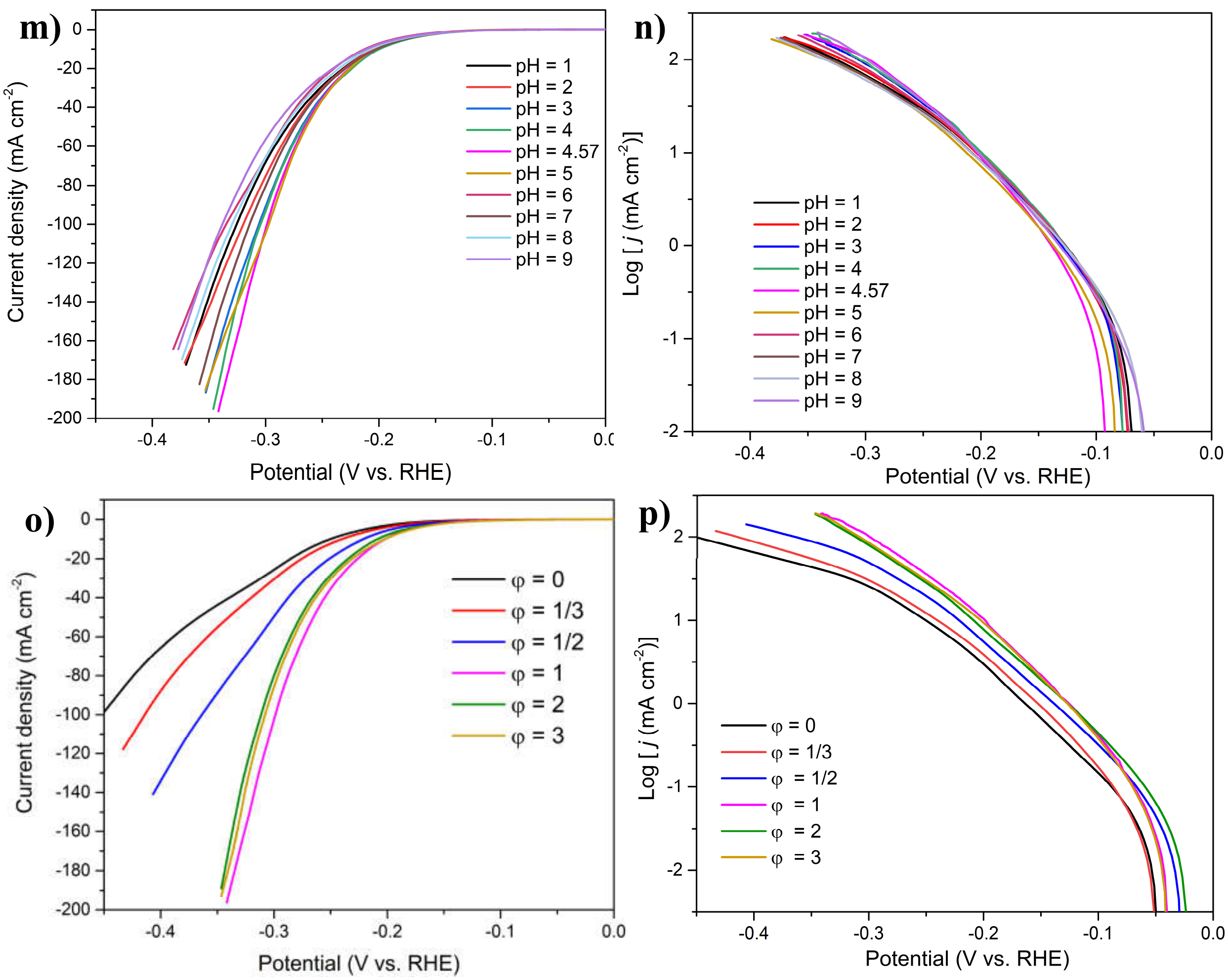

Figure S1. LSV and Tafel plots of the effect of (a,b) Nafion loading, (c,d) electrocatalyst ($NiMoO_4$) loading, (e,f) carbon black loading, (g,h) calcination temperature, (i,j) calcination time, (k,l) Ni/Mo atomic ratio, (m,n) precursor solution's pH, and (o,p) fuel-to-oxidant ratio (φ). Measurements were conducted in 0.5 M H_2SO_4 at a scan rate of 1 mV s^{-1} and corrected for ohmic (*iR*) drop. The electrocatalyst layer consisted of $NiMoO_4$, carbon black (XC-72R) and Nafion except for (k,l) which consisted of Ni_xMo_{1-x}-oxide ($0 \leq x \leq 1$).

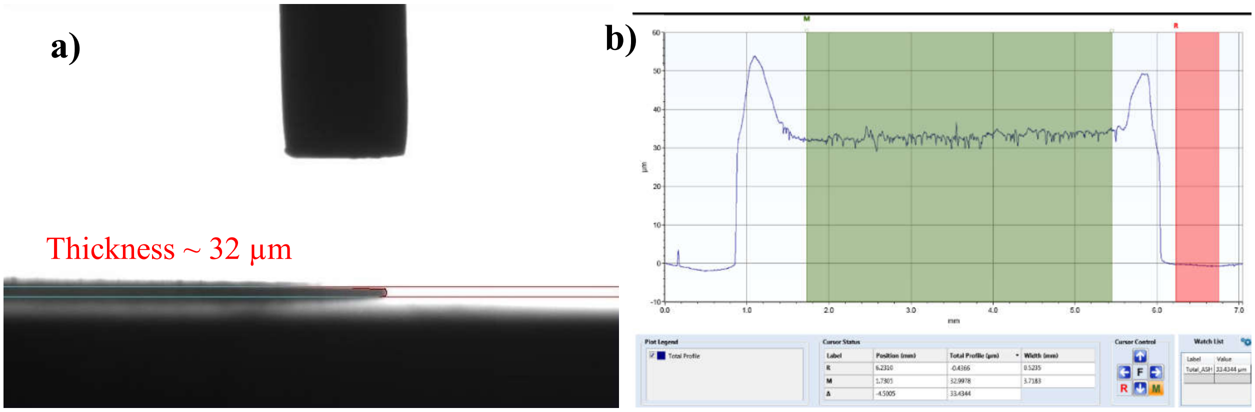

Figure S2. (a) A side-view representation of the $NiMoO_4$/CB/Nafion electrocatalyst layer (same specifications as those indicated in the caption of Figure 5 in the main text) portraying its thickness, (b) a topography profile used to obtain the thickness of the catalytic layer. Both methods converge with the goniometer and profilometer yielding 32 µm and 33 µm, respectively.

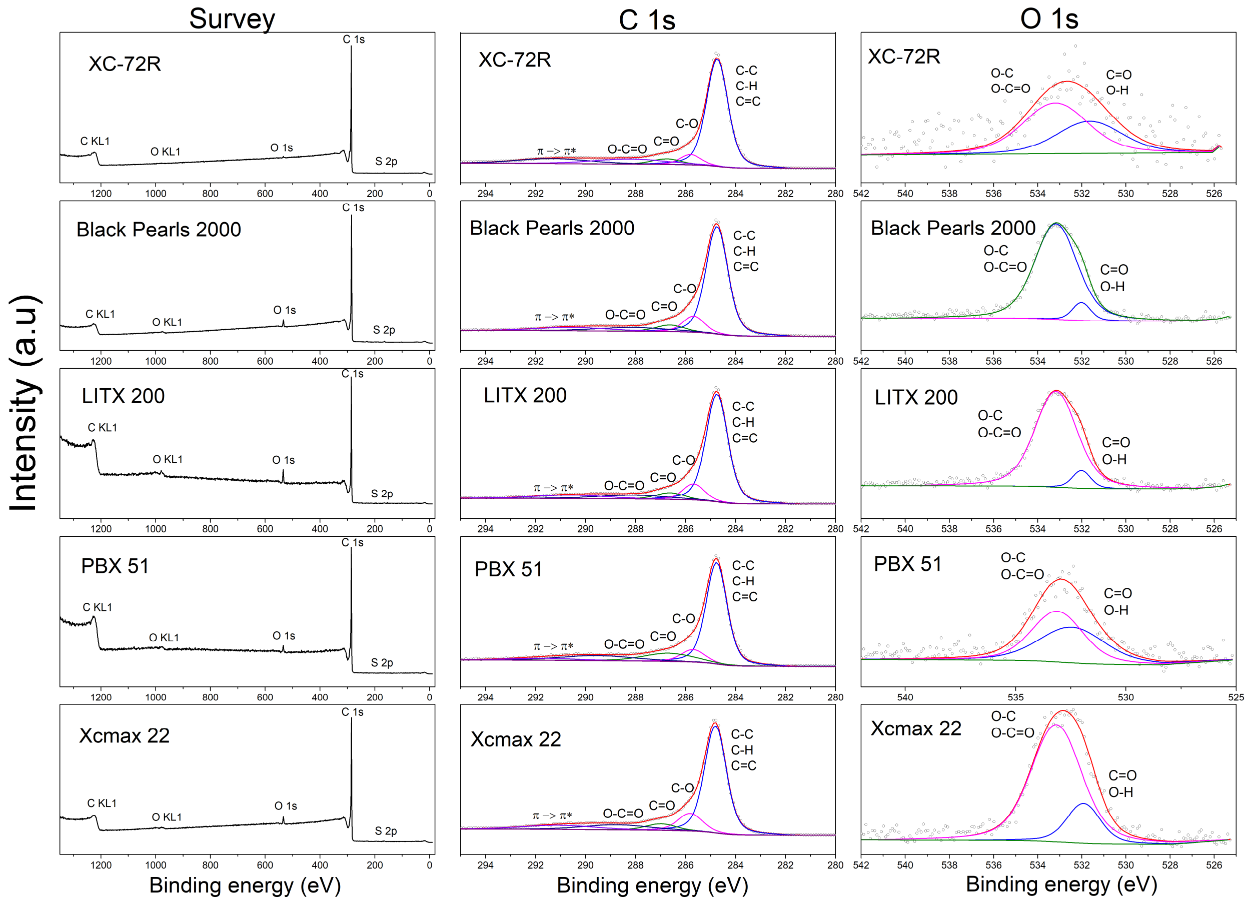

Figure S3. XPS survey, and high-resolution C1s and O 1s spectra of different types of carbon black (XC-72R, Black Pearls 2000, LITX 200, PBX 51, and Xcmax 22).

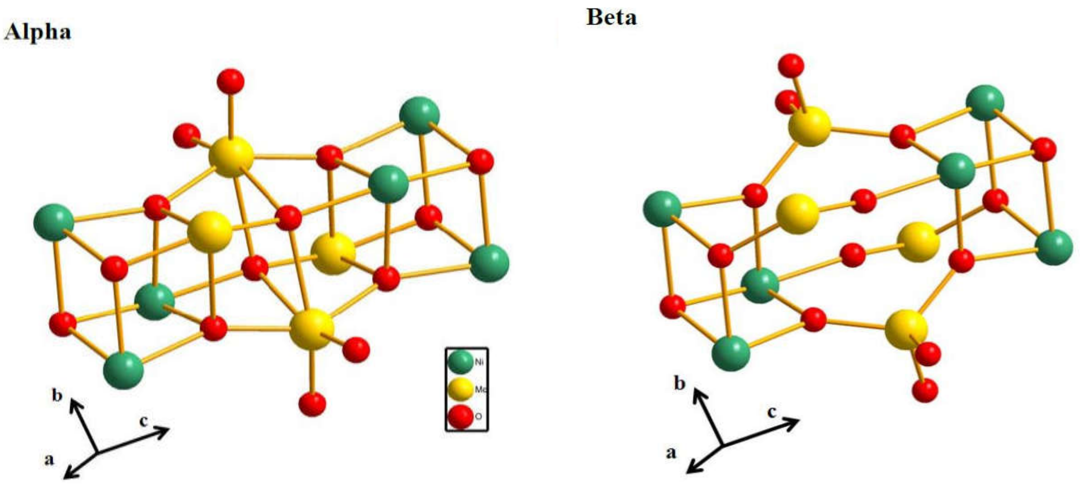

Figure S4. Partial view representation of α-$NiMoO_4$ and β-$NiMoO_4$ polymorphs [1].

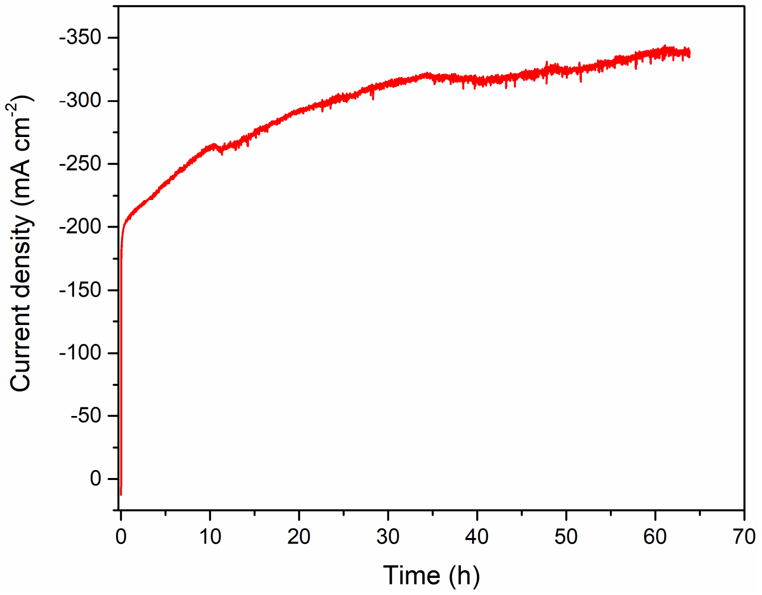

Figure S5. Long-term HER (water electrolysis) test employing the $NiMoO_4$/CB/Nafion electrocatalyst layer (same specifications as those indicated in the caption of Figure 5 in the main text) as the working electrode (cathode) and a Pt coil as the counter electrode. The performance is clearly ramping as the experiment progresses due to the dissolution of Pt and the migration of its ions to the cathodic side, where they get electrodeposited on the working electrode improving its electrocatalytic HER performance.

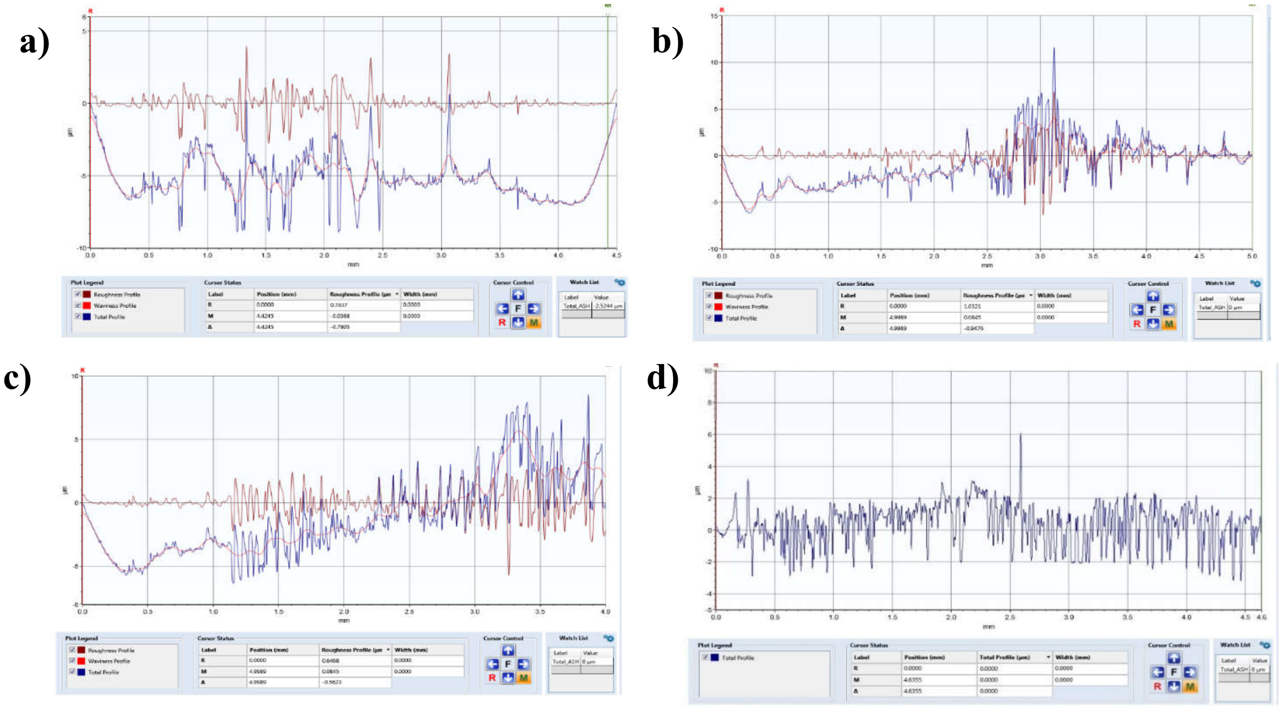

Figure S6. Profilometry measurements of the $NiMoO_4$/CB/Nafion electrocatalytic layer (same specifications as those indicated in the caption of Figure 5 in the main text) (a) before and after (b) 15 min, (c) 1 h, and (d) 24 h of water electrolysis in 0.5 M H_2SO_4 at an overpotential of -0.58 V.

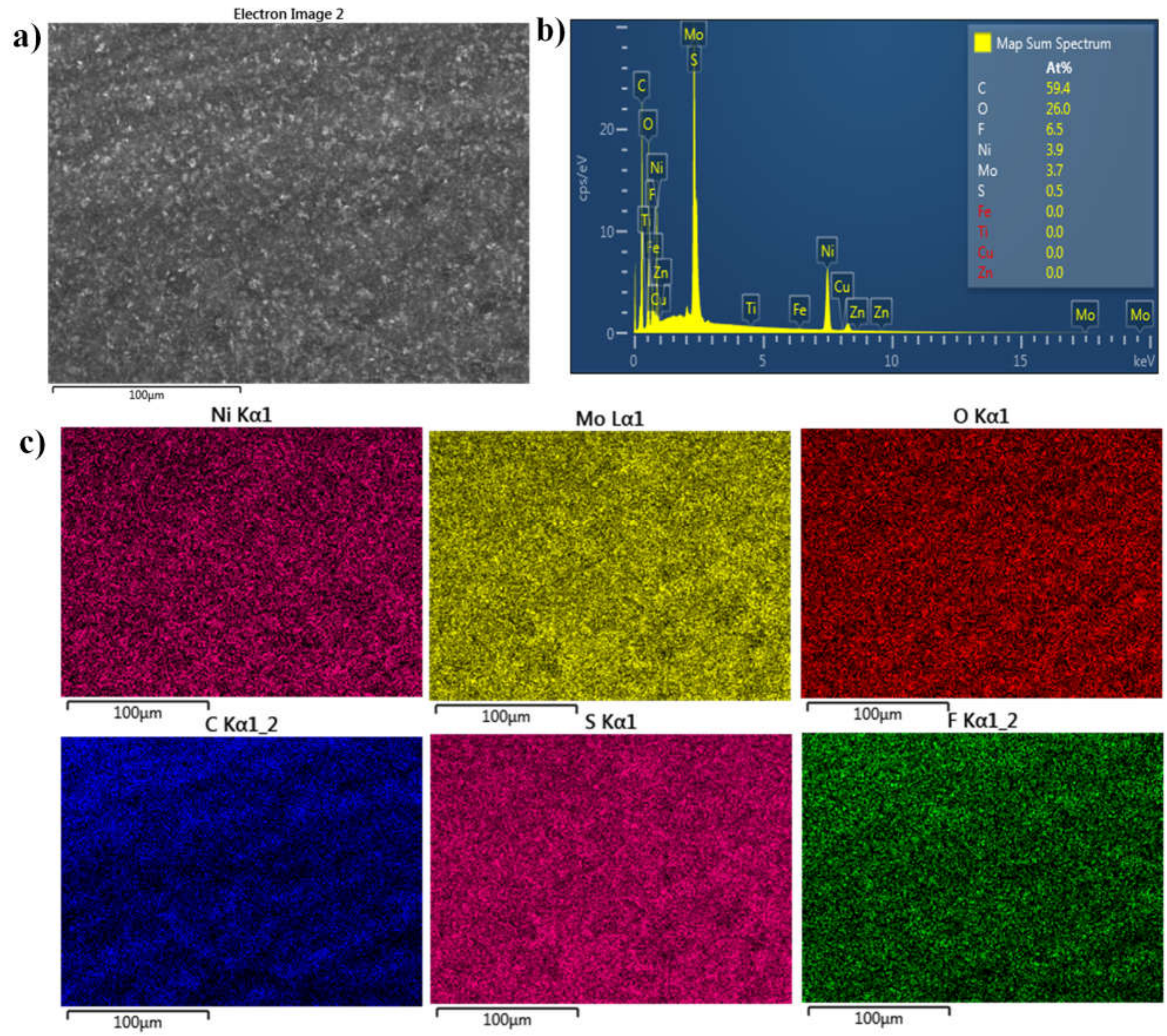

Figure S7. EDX analysis of the $NiMoO_4$/CB/Nafion electrocatalyst layer (same specifications as those indicated in the caption of Figure 5 in the main text) before the 24-hour electrolysis test in 0.5 M H_2SO_4 at an overpotential of -0.58 V showing the a) SEM images over which the analysis was conducted, b) elemental composition of the elements of interest, and c) their respective mapping distribution.

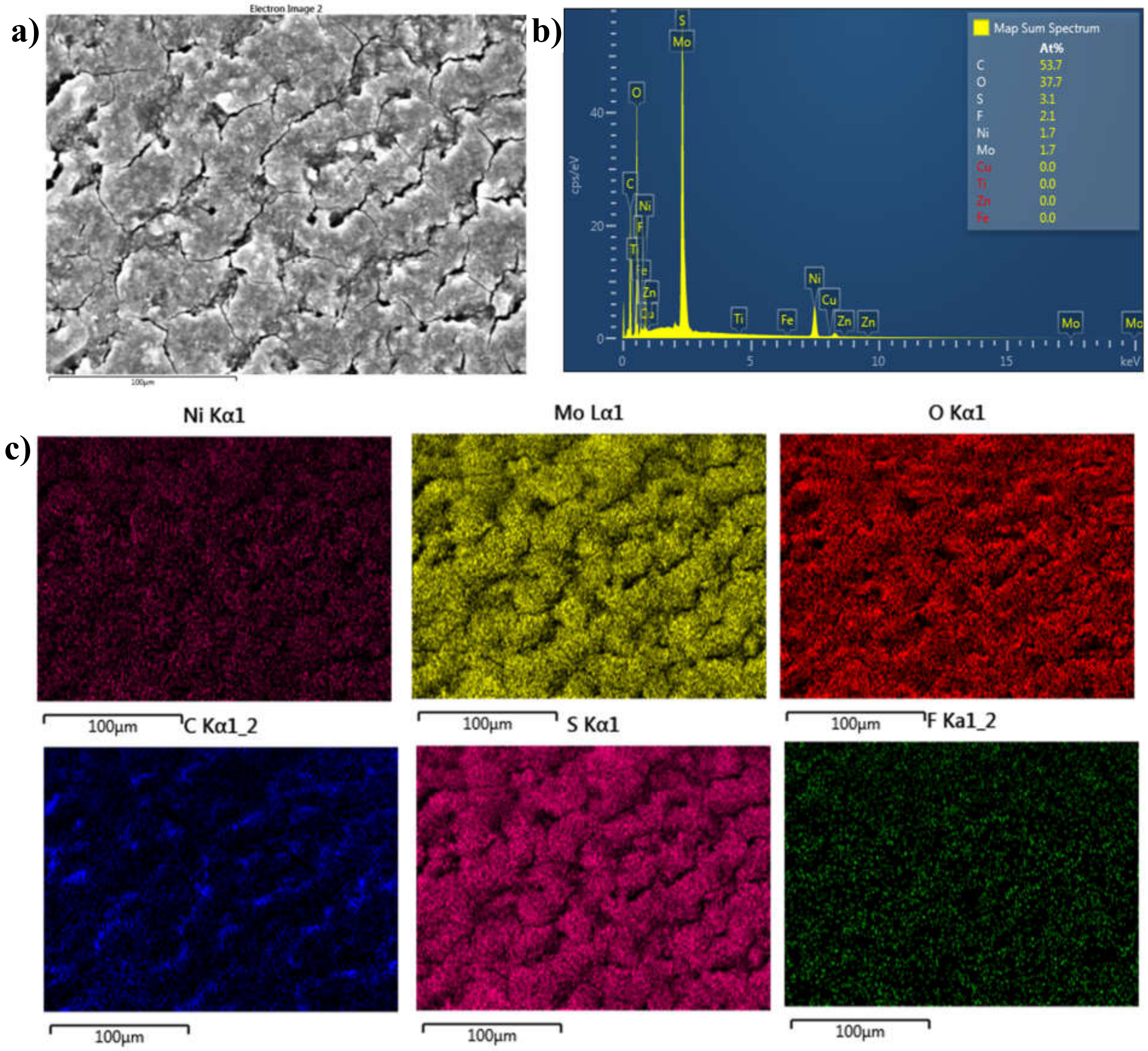

Figure S8. EDX analysis of the $NiMoO_4$/CB/Nafion electrocatalyst layer (same specifications as those indicated in the caption of Figure 5 in the main text) after the 24-hour electrolysis test in 0.5 M H_2SO_4 at an overpotential of -0.58 V, showing the a) SEM images over which the analysis was conducted, b) elemental composition of the elements of interest, and c) their respective mapping distribution.

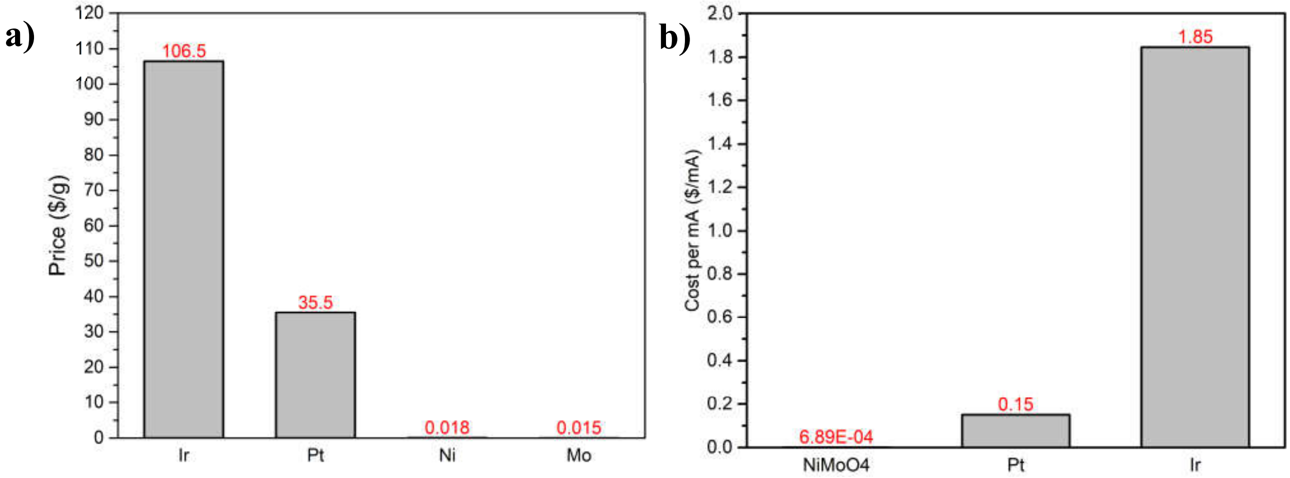

Figure S9. a) The current market price of Ir, Pt, Ni, and Mo in US dollars [2], b) the cost normalized HER performance of the material.

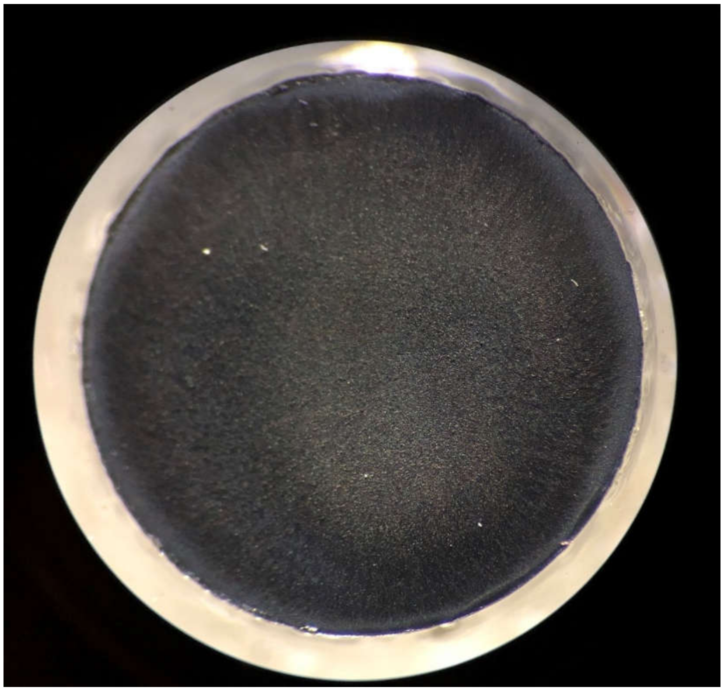

Figure S10. An optical microscopy representation of the $NiMoO_4$/CB/Nafion electrocatalyst layer (same specifications as those indicated in the caption of Figure 5 in the main text, magnification 10x).

1. Théodet, M., *New generation of" bulk" catalyst precursors for hydrodesulfurization synthesized in supercritical fluids*. 2010, Université Sciences et Technologies-Bordeaux I.
2. *Commodity and metal prices*. January 2021]; Available from: https://go.nature.com/2Zy1HeJ
